# Modular Use of the Uniquely Small Ring A of Mersacidin Generates the Smallest Ribosomally Produced Lanthipeptide

**DOI:** 10.1021/acssynbio.2c00343

**Published:** 2022-09-06

**Authors:** Jakob H. Viel, Oscar P. Kuipers

**Affiliations:** Department of Molecular Genetics, University of Groningen, Nijenborgh 7, 9747 AG Groningen, The Netherlands

**Keywords:** mersacidin, lanthipeptide, E. coli, engineering, RiPPs, peptide

## Abstract

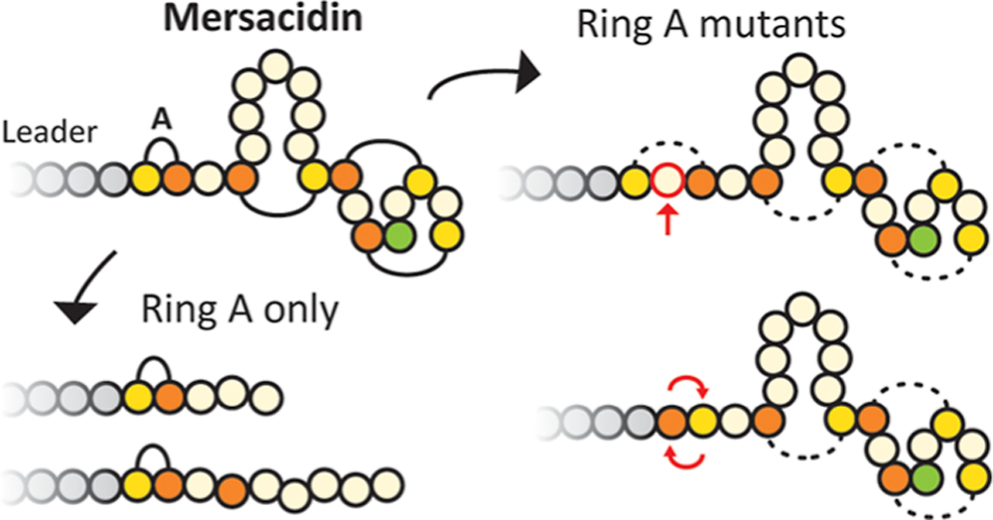

Mersacidin is an antimicrobial class II lanthipeptide. Lanthipeptides are a class of ribosomally synthesized and post-translationally modified peptides (RiPPs), characterized by intramolecular lanthionine rings. These rings give lanthipeptides their bioactive structure and stability. RiPPs are produced from a gene cluster that encodes a precursor peptide and its dedicated unique modification enzymes. The field of RiPP engineering aims to recombine modification enzymes from different RiPPs to modify new substrates, resulting in new-to-nature molecules with novel or improved functionality. The enzyme MrsM from the mersacidin gene cluster installs the four lanthionine rings of mersacidin, including the uniquely small ring A. By applying MrsM in RiPP engineering, this ring could be installed in linear peptides to achieve stabilization by a very small lanthionine or to create small lanthionine-stabilized modules for chemical modification. However, the formation of unique intramolecular structures like that of mersacidin’s ring A can be very stringent. Here, the formation of ring A of mersacidin is characterized by mutagenesis. A range of truncated mersacidin variants was made to identify the smallest possible construct in which this ring could still be formed. Additionally, mutants were created to study the flexibility of ring A formation. It was found that although the formation of ring A is stringent, it can be formed in a core peptide as small as five amino acids. The truncated mersacidin core peptide CTFAL is the smallest ribosomally produced lanthipeptide reported to date, and it has exciting prospects as a new module for application in RiPP engineering.

## Introduction

**Figure 1:**
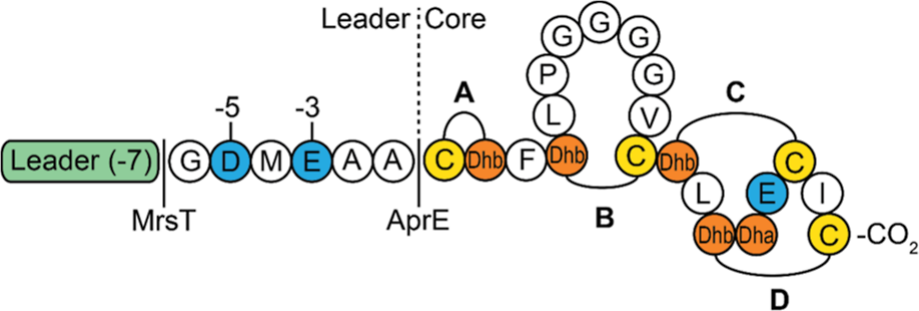
Fully modified mersacidin with leader peptide. The fully modified core peptide has four lanthionine rings, of which ring A is uniquely small.^1^ The negatively charged leader peptide residues D-5 and E-3 are crucial for the formation of one specific ring, which is most likely ring A. The rings installed by MrsM, both very small ring A and large ring B, and the nonleader-dependent decarboxylation by MrsD, are both fundamentally interesting and useful for the application in RiPP engineering. Fully modified MrsA is processed in two steps, MrsT cleaves before residue G-6 before export,^13^ after which AprE cleaves the remaining six amino acids in the supernatant.^16^ Dha: dehydroalanine, dehydrated serine residue. Dhb: dehydrobutyrine, dehydrated threonine residue.

Mersacidin is a class II lanthipeptide produced by *Bacillus amyloliquefaciens*,^1^ which has good antimicrobial activity against a range of Gram-positive bacteria, including methicillin-resistant *Staphylococcus aureus* strains^1−3^ (Figure 1). Lanthipeptides are a class of ribosomally synthesized and post-translationally modified peptides (RiPPs).^4^ RiPPs comprise a large family of ribosomally synthesized peptides, which are modified by dedicated enzymes after translation to obtain their bioactive form. Because of their ribosomally synthesized nature, the application of synthetic biology methods to recombine and improve RiPPs with the purpose of creating new antimicrobial compounds has grown into a useful and rapidly expanding field of research.^5,6^ On RiPPs and RiPP engineering, there are some excellent recent reviews available.^4,6^

Lanthipeptides are characterized by their intramolecular thioether rings, which are formed between cysteine residues, and dehydrated serine or threonine residues.^7,8^ These rings give lanthipeptides their bioactive structure and also make them more resistant to thermal and proteolytic degradation.^9^ Because the lanthionine rings of mersacidin are installed by a single LanM enzyme, MrsM, mersacidin is classified as a class II lanthipeptide.^4^

**Figure 2:**
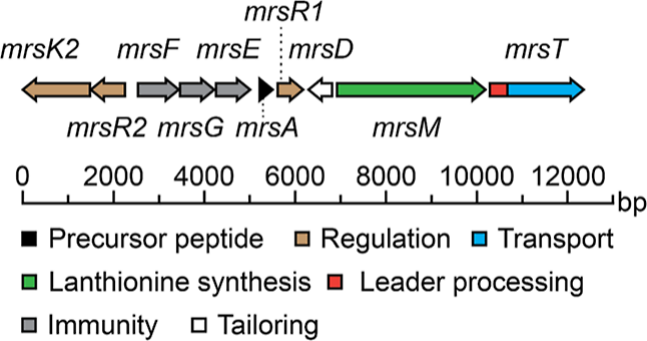
Mersacidin biosynthetic gene cluster. The two-component system MrsK2R2 regulates the expression of immunity genes MrsFGE.^11^ MrsR1 regulates the biosynthetic genes.^11^ The precursor peptide MrsA is modified by MrsMD,^12−14^ after which it is exported by the bifunctional transporter and leader protease MrsT, which also partially cleaves the leader upon transport.^12,13^

The mersacidin gene cluster encodes the precursor peptide and nine enzymes that perform the functions of post-translational modification, regulation,^10,11^ host self-immunity,^12^ transport,^12^ and partial leader processing, respectively^12,13^ (Figure 2). The precursor peptide MrsA is translated as a linear peptide, consisting of an N-terminal leader peptide and a C-terminal core peptide, of which the decarboxylase MrsD removes the CO_2_ from its C-terminal cysteine residue.^14,15^ Then, the lanthionine synthetase MrsM dehydrates the serine and threonine residues to dehydroalanine (Dha) and dehydrobutyrine (Dhb), respectively, and subsequently installs lanthionine rings from the Dhb residues to specific cysteine residues^12,13^ (Figure 1). When fully modified, the peptide is transported out of the cell by MrsT,^12^ which cleaves the leader peptide up to six residues from the core peptide.^13^ Finally, the nonspecific extracellular *B. amyloliquefaciens* protease AprE cleaves off the remaining residues of the leader peptide, releasing bioactive mersacidin.^16^

Recently, a heterologous expression system for mersacidin in *Escherichia coli* has been developed, which requires only three biosynthetic genes.^13^ In this system, genes that encode a His-tagged precursor peptide, His6-MrsA, and the modification enzymes MrsMD are coexpressed to produce fully modified His6-MrsA. After removing the leader peptide with the heterologously expressed His-tagged protease AprE-His, bioactive mersacidin is obtained.^16^ This system has already been applied to study the mersacidin leader, identifying the Asp -5 and Glu -3 residues to be crucial for specific ring formation, thereby elucidating the function of the two-step leader processing of mersacidin.^17^ A next step in the application of the heterologous expression system is the characterization of interesting mersacidin modifications, yielding modules for use in RiPP engineering.

Several RiPP engineering methods have been described that would greatly benefit from the application of mersacidin modifications. The C-terminal decarboxylation performed by the leader-independent tailoring enzyme MrsD and the large ring B of mersacidin already offer valuable opportunities for RiPP engineering. However, the greatest prospect of mersacidin modifications in RiPP engineering lies in the application of the uniquely small ring A.

Ring A of mersacidin could, for example, be installed into linear therapeutic peptides to increase their stability and thereby their therapeutic value.^18^ Because installing a lanthionine ring into a bioactive peptide can interfere with their biological function, the uniquely small size of ring A makes it especially suitable for the stabilization of such peptides. Additionally, ring A might be a suitable replacement for Pro, X-Pro, or Pro-X^19^ sequences since it is expected to make a structural kink in the chain.

Another very interesting future application for this smallest ring A could be its application as a stable module for chemical modifications. It has recently been shown that by adding functional groups, for example, fatty acid chains, to otherwise nonactive lanthipeptides, good antimicrobial activity can be obtained.^20^ Lanthipeptides or fragments thereof, can in this sense, be used as a stable module to which all kinds of modifications can be added in vitro. The proposed ring A module could be obtained through the incorporation of noncanonical amino acids that have convenient chemical handle side chains. For example, the methionine analogues, azidohomoalanine (Aha) and homopropargylglycine (Hpg), which are used in click chemistry.^21^ Another, perhaps more straightforward approach is the addition of chemical handles to the purified peptide, which can be directed to the peptide’s C-terminal carboxyl group or negatively charged residues.^20,22^ The small size of ring A of mersacidin could also be beneficial here, as the stabilizing lanthionine module could interfere with the biological activity of any clicked moieties. However, it is not certain that ring A can be formed without the rest of the mersacidin peptide.

Ring A of mersacidin has unusual characteristics. It is formed between the dehydrated Thr2 and its upstream Cys1 residue (Figure 1).^12,23^ This is in contrast with the other three rings of mersacidin, which are formed from dehydrated Thr to downstream Cys residues. Ring A also spans no additional amino acids and likely needs specific residues of the leader peptide to be formed.^17^ Because of these unusual characteristics ring A has stringent formation conditions.^23^ For this reason, the characterization of ring A formation prerequisites would greatly aid its application success in RiPP engineering. To determine the minimal size of a peptide containing ring A of mersacidin, a range of truncated mersacidin mutants was produced using the heterologous expression system for mersacidin in *E. coli*.^13^ These peptides, with decreasing length and number of possible rings, are coexpressed with MrsM to determine the smallest possible ring A containing construct.

To characterize the flexibility of formation, an additional range of ring A mutants was created with a varying ring size and reversed direction of formation. The combined information on the minimal ring A construct size and flexibility of ring A formation gives useful insights into the prospects of its application in RiPP engineering.

## Results and Discussion

### Truncated Mersacidin Variants

To determine the minimal size of a construct containing ring A of mersacidin, a range of truncated mersacidin mutants was created (Table 1) and coexpressed with MrsM in *E. coli*.^13^ For straightforward analysis, the relatively complex region Thr13–Cys20 was completely removed in all truncated constructs. Construct *a*, which deviates the least from wildtype mersacidin, can theoretically form ring A and ring B. The other truncated constructs gradually decrease in number of possible post-translational modifications and peptide length. All of the constructs were purified by Ni-NTA chromatography, and the expression yield of each construct was determined by tricine SDS-page to be 1–15 mg/L of expression volume (**S7**). The Ni-NTA purified peptides were separated by HPLC (**S3**, **S4**), after which promising peptides from candidate peaks were analyzed by LC–MS. Additionally, LC–MS runs of TCEP-reduced samples and iodoacetamide (IAA) free-cysteine assays were used to detect the presence of any disulfide bridges and to confirm lanthionine ring formation. The IAA free cysteine assays result in a 57 Da addition to the peptide’s mass per free cysteine.

**Table 1:**
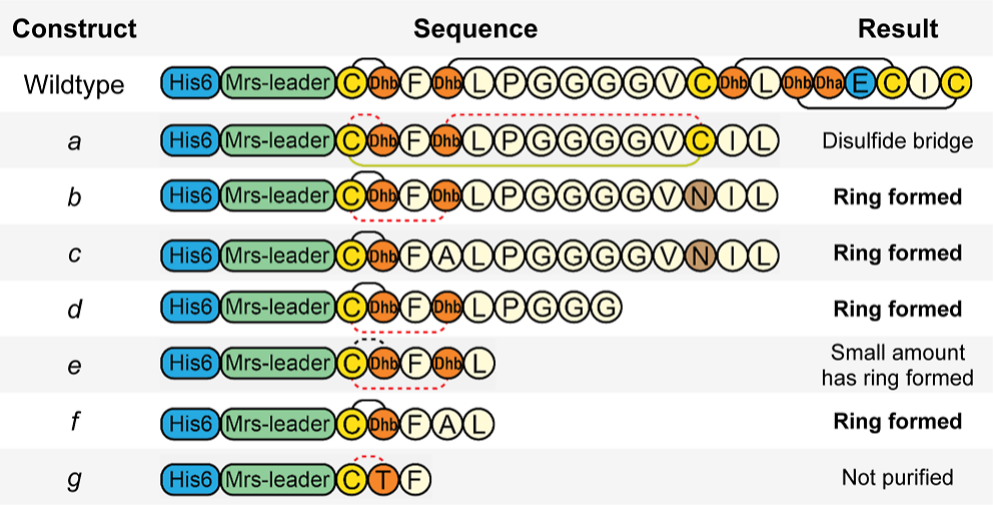
Truncated Mersacidin Mutants^a^

a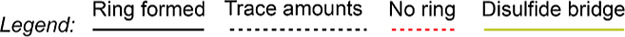.

**Figure 3:**
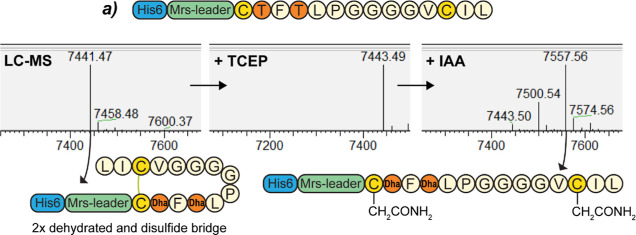
LC–MS and free cysteine assay of purified truncated mersacidin construct *a*. The product isolated from HPLC purification resembles the mass of the fully dehydrated peptide −2 Da (7441.47, 7443.49 Da theoretical). Reduction by TCEP increases the mass to the theoretical mass, meaning a disulfide bridge naturally forms in this construct, and at most small amounts of product contain lanthionine rings (**S4**).

For construct *a* (Table 1), one large candidate peak was collected from HPLC purification and analyzed by LC–MS (Figure 3, **S4**). While this peptide can theoretically be dehydrated twice and contain two lanthionine rings, the measured monoisotopic mass of the peptide was 2 Da lower than the twice dehydrated theoretical mass (7441.47, 7443.49 Da theoretical), pointing toward the formation of a disulfide bridge. After reducing the peptide with TCEP, the peak shifted 2 Da toward the theoretical mass, confirming the formation of a disulfide bridge in the large majority of all products (Figure 3). A free cysteine assay was performed on the TCEP-reduced peptide, shifting nearly all products away from the theoretical mass, in line with the unmodified control (**S4**). While the complete absence of modified product cannot be confirmed, only trace amounts (at most) of the modified product are produced in the case of construct *a*. Interestingly, the yield of construct *a* was lower than that of all of the well-modified truncated constructs (**S7**). This indicates that the disulfide bridge of construct *a* offers less protection from degradation than the lanthionine rings in other constructs. The lack of ring formation in this construct is notable because the amino acid sequence of the first two rings of mersacidin is completely intact, and the formation of at least ring A should not be impaired. Previously, the possible formation of a disulfide bridge was also reported when negatively charged residues Asp-5 and Glu-3 were removed from the mersacidin leader sequence.^17^ Mutation of those residues most likely affected the dehydration of Thr2.^17^ Taking these results together, it appears that in mersacidin maturation, the formation of ring A and ring B are mechanistically interdependent. In this case, closing of ring A may depend on the prior formation of ring B, which is discussed later. However, as the construct tested here was fully dehydrated, it is not clear why MrsM was unable to close either ring.

**Figure 4:**
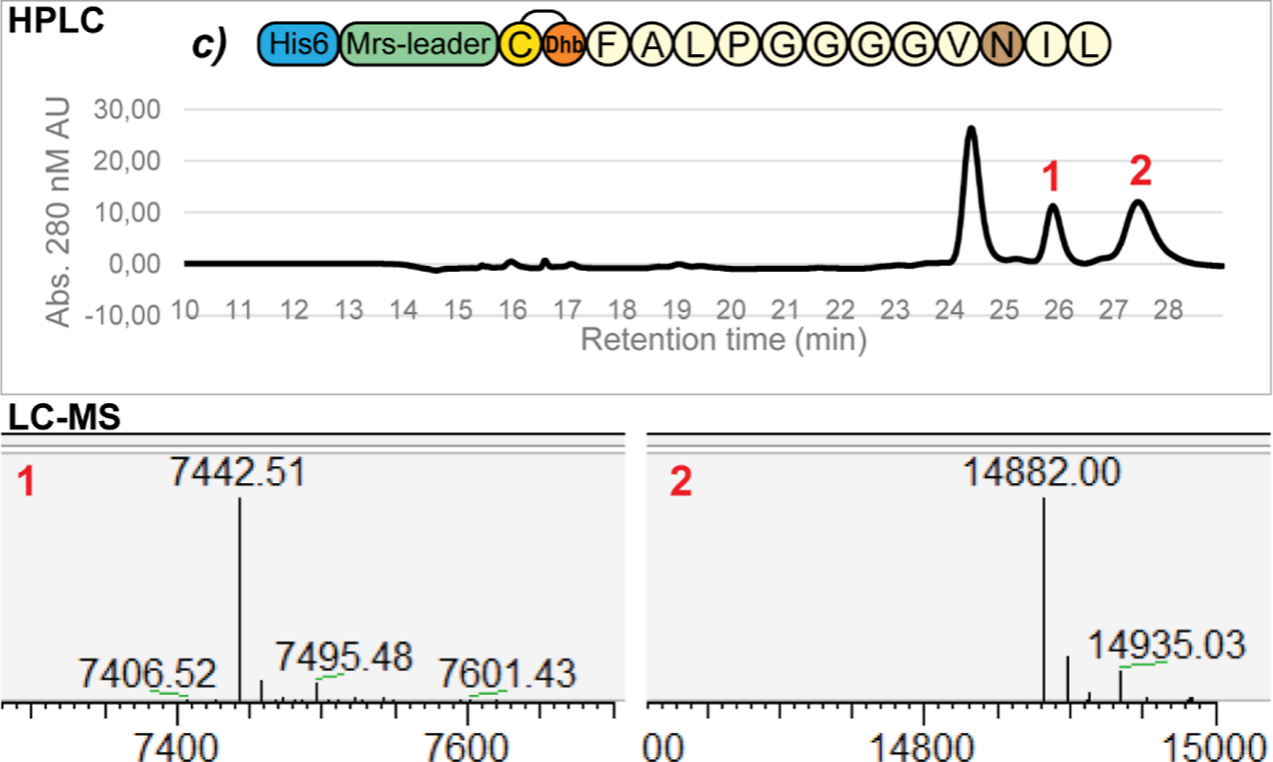
HPLC and LC–MS spectra of construct *c*. The Thr4Ala and Cys12Asn substitution results in much lower modification heterogeneity, and the substitution of Cys12 prevents the formation of an intramolecular disulfide bridge. In the product under peak 1, the ring is installed successfully (7442.51, 7442.53 Da theoretical) (**S4**). However, the product under peak 2 is dehydrated, but it is dimerized through intermolecular disulfide bonds. TCEP reduction of this peak and a subsequent free cysteine assay reveals the product is fully dehydrated but contains no lanthionine ring (**S4**).

**Figure 5:**
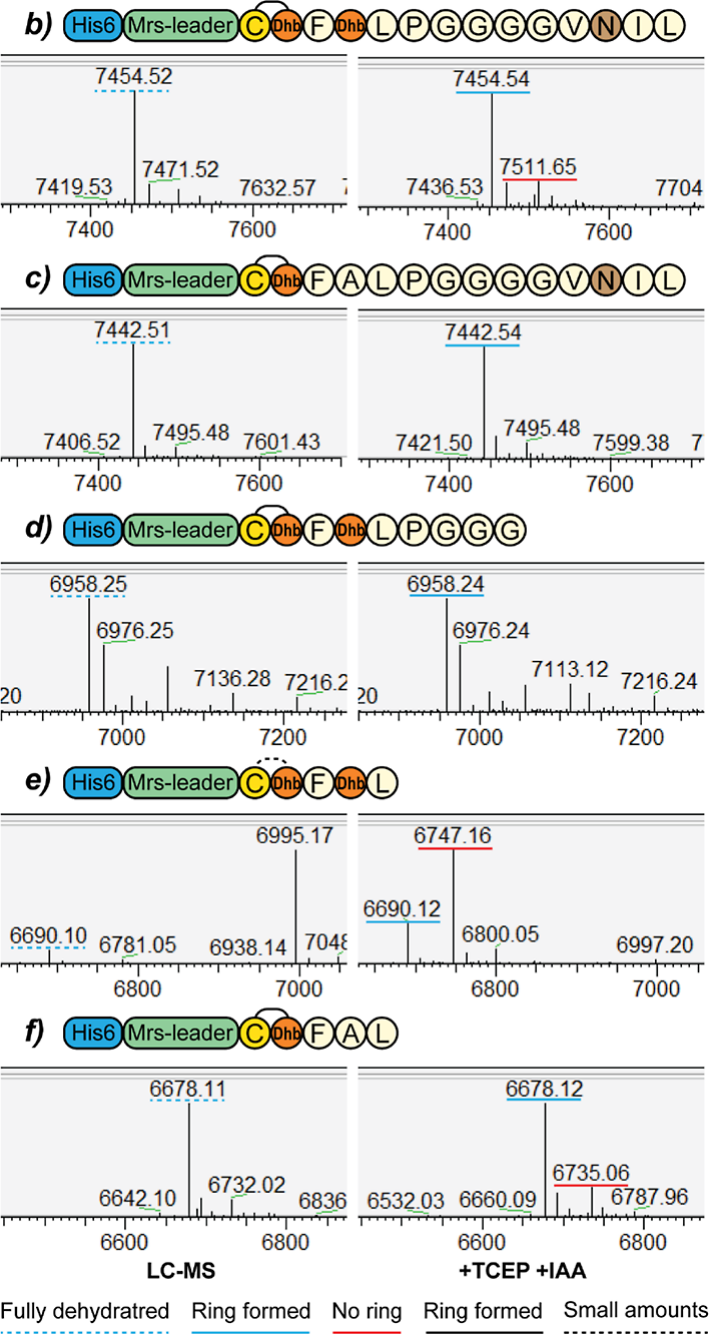
LC–MS and free cysteine assays of the best modified HPLC fraction per truncated mersacidin variant. An overview of the best modified products, purified by HPLC for each of the constructs, showing the fully modified mass (underlined blue) and the mass shifts resulting from the free cysteine assays (underlined red). In general, the mutants containing both Thr2 and Thr4 have a lower modification efficiency than those containing only Thr2. The modification efficiency of construct *c* is better than that of construct *b*, and the modification efficiency of construct *f* is much better than that of construct *e*. The fractions from constructs *c* and *d* contain only fully modified products. However, construct *c* still has a quite long amino acid sequence, and construct *d* has a higher modification heterogeneity due to its two Thr residues. Construct *f* is very short, has low modification heterogeneity, and its ring is formed in almost all dehydrated products. For these reasons, it is the most promising candidate for further optimization and application (S4, S6).

In construct *b* and *c*, Cys12 was replaced by an Asn residue, which makes the formation of an intramolecular disulfide bridge impossible, and construct *c* has an additional Thr4Ala substitution (Table 1, Figure 4). Both construct *b* and *c* were produced in relatively good amounts (**S7**), and their purification yielded fractions containing fully modified peptides. For construct *b*, the theoretical mass resembled the observed mass (7454.52, 7454.53 Da theoretical), and the free cysteine assay resulted in a 57 Da shift for a small fraction of the product (Figure 5), meaning that the two possible dehydrations and single lanthionine ring are installed in the majority of the product.

For construct *c*, two candidate peaks could be isolated (Figure 4, **S4**). The first peak contained a fully dehydrated and cyclized peptide (7442.51, 7442.53 Da theoretical). This product appears to have a better modification efficiency than construct *b* (Figure 5). Interestingly, the second peak contained a dimer of dehydrated uncyclized peptide (Figure 4, S4), which could be reduced by TCEP to yield the expected product mass (14882.00 Da reduced to 7442.52, 7442.53 Da theoretical).

When comparing construct *b* to construct *c*, the Thr4Ala substitution results in less heterogeneity and more complete modification (Figure 4, S4). Additionally, the peak of interest in the HPLC spectrum of construct *c* is more isolated and can therefore be purified more easily.

For construct *d*, which is truncated inside the glycine motif of ring B, three separate candidate peaks were isolated by HPLC. The smallest peak, with the lowest retention time, contained the peptide with both one and two dehydrated threonines (−2H_2_O = 6958.25, 6958.23 Da theoretical; −1H_2_O = 6976.25, 6976.24 Da theoretical) (Figure 5, S4). The ring was formed in both the single and double dehydrated peptides from this fraction. This shows that ring A can be formed without prior dehydration of Thr4 although ring formation efficiency seems to be generally higher when this residue is mutated to an Ala residue in constructs *c* and *f*.

The product from the second HPLC peak contained mostly uncyclized peptides, while the third candidate peak contained an unknown degradation product (**S4**). While construct *d* has a good expression yield (**S7**) and efficient ring formation in part of the product, the heterogeneity resulting from the single and double dehydration states makes this construct less attractive for ring A applications.

In constructs *e* and *f*, the core peptide is reduced to the first five amino acids of the mersacidin core, in which Thr4 is replaced by an Ala residue in construct *f*. Construct *e* was produced at around the same high level as that of construct *d* (**S7**). HPLC purification of construct *e* resulted in two candidate peaks (**S4**). The major product from peaks one and two contained 1 × dehydrated peptide (−1H_2_O = 7013.18, 6708.12 Da theoretical) and 2 × dehydrated peptide (−2H_2_O = 6995.17, 6690.11 Da theoretical), respectively, both with an unknown adduct of ca. 305 Da. Peak two also contains some product of the expected 2 × dehydrated mass, of which the majority shifted in the free cysteine assay, confirming the results from construct *d* that although Thr4 does not necessarily needs to be dehydrated for ring A to form, its dehydration does considerably increase ring A formation. While the free cysteine assays of peak two indicate that some fully mature peptide can be formed, the low modification efficiency and presence of unknown adducts make construct *e* not suitable for application.

HPLC purification of construct *f* resulted in two products of interest (**S4**), one of which contained the fully dehydrated product that showed almost no shift in the free cysteine assay (6678.11 Da, 6678.11 theoretical) (Figure 5). The substitution of the Thr4 to an Ala residue in construct *f* is thus the solution to avoid poor modification efficiency in these truncated variants, which was also seen in the comparison between mutants *b* and *c*. Not only does this substitution leads to more efficient ring formation, but it also results in easier separation of peaks by HPLC and lower heterogeneity of products underneath the peaks. The previously mentioned characteristics combined with the decent expression level (**S7**) of this variant make this truncated variant an attractive candidate for future application.

Finally, the smallest construct, *g*, of which the core peptide comprises only the amino acids CTF, could not be purified by HPLC. The peptide is degraded during the long expression protocol (**S7**), indicating that the threonine in this construct is not dehydrated and that MrsM needs more than one amino acid downstream of Thr2 for dehydration to occur. Three amino acids downstream of Thr2 are shown to work well in construct *f*, and although not tested here, a length of two amino acids might also be sufficient.

The leader peptide of all mersacidin variants was removed with AprE-His,^16^ after which their antimicrobial activity against *Micrococcus flavus* was tested (**S8**). For none of the constructs, antimicrobial activity could be detected.

In conclusion, construct *f* is the most suitable construct for utilization in RiPP engineering. Its small size, simple ring topology, decent modification efficiency, and good production yield make this an attractive candidate for future application.

### Ring A Mutants

To investigate the flexibility of ring A formation, a range of ring A mutants was expressed and analyzed by LC–MS (Table 2). The constructs *h* and *i*, where Thr2 was substituted with an Asn and an Ala residue, respectively, function as the negative control for lack of dehydration in ring A. LC–MS of these mutants showed that they are dehydrated maximally three out of four times (**S5**). This is a notable result, as the formation of a maximum of three dehydrations was also reported when the negatively charged residues were removed from the GDMEAA sequence of the mersacidin leader.^17^ The lack of ring A formation prevents the dehydration of another Thr residue in the peptide. This result indicates that the lack of two dehydrations observed when the GDMEAA sequence is removed results from Thr2 of Ring A not being dehydrated, preventing its cyclization. This in turn, prevents the dehydration of another Thr residue in the peptide.

**Table 2:**
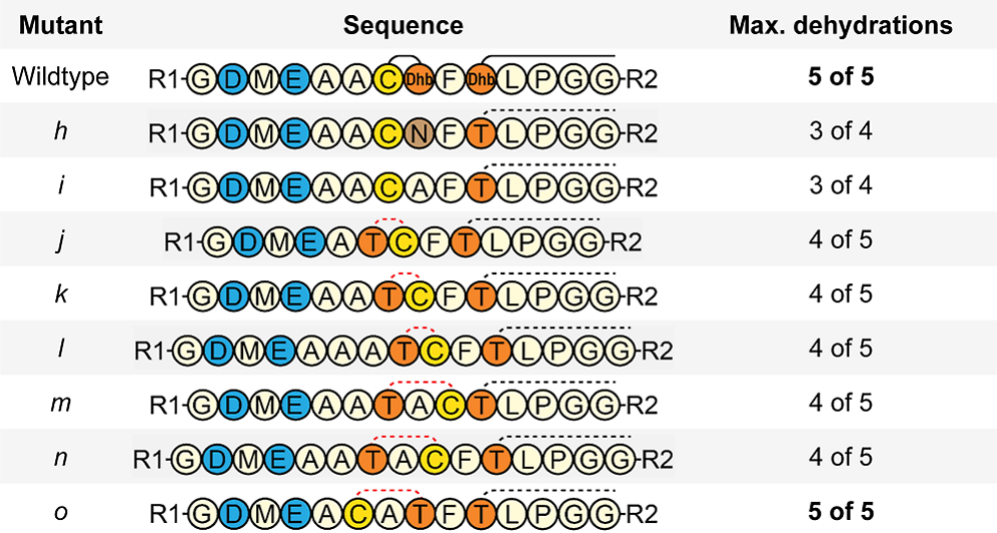
Mutants of Ring A^a^

a R1 = His6-mersacidin −48 to −7, R2 = mersacidin 9 to 20. 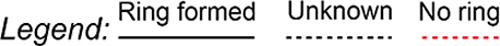.

**Figure 6:**
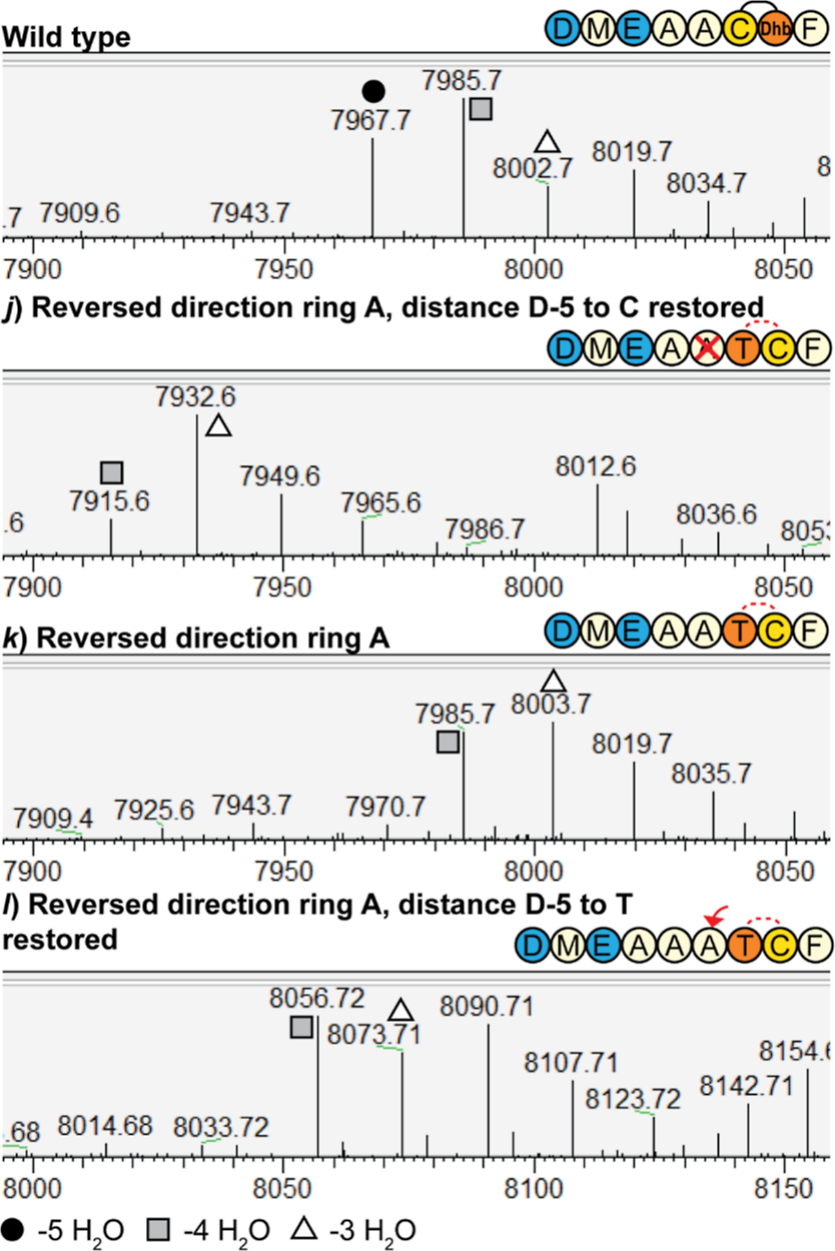
LC–MS of reversed ring A mersacidin variants. LC–MS analysis was performed on the constructs where the ring A formation direction was reversed. Although in all of the constructs, the maximum number of dehydrations occurring is four out of five, there are differences in the modification efficiency between the different constructs. The lowest modification efficiency is seen for construct *j*, where the distance from Asp-5 to Cys2 is restored to the original distance. For this construct, the majority of the product is dehydrated three times. In construct *k*, the ring direction is reversed without altering the leader sequence, which leads to a higher modification efficiency. Restoring the distance from Asp-5 to Thr1 in construct *l* appears to slightly increase the modification efficiency, but in none of the constructs, five dehydrations could be installed. The uniquely small ring A of mersacidin can thus not be formed in the same direction as its other three rings.

Next, the effect of reversing the direction of ring A was tested (Table 2*jkl*, Figure 6). Since the distance from the mersacidin leader residues Asp-5 and Glu-3 to ring A is crucial for ring formation,^17^ constructs were made to restore the distance of these residues to the Cys (*j*) or Thr (*l*) residue of ring A, in addition to the construct only reversing the ring (*k*). Neither of these constructs was dehydrated more than four times, although the construct restoring the distance of Asp-5 and Glu-3 to ring A’s Thr residue (*l*) is dehydrated much more efficiently than the construct restoring the distance of Asp-5 and Glu-3 to the Cys residue (*j*) (Figure 6).

This result indicates that the Thr1 residue can still be efficiently dehydrated without forming a ring, especially when the original distance to Asp-5 and Glu-3 is maintained. The lack of ring A formation then prevents the dehydration of a downstream residue, as is observed in the control constructs *h* and *j*. Alternatively, a different ring topology could be present in constructs *jkl*.

Constructs *m* and *n*, where ring A direction is reversed and where the ring size is increased by one through the addition of an Ala residue, showed the same dehydration pattern as the construct where the ring size was not increased (*k*) (**S5**). However, this result is in line with the observation that the distance of Asp-5 and Glu-3 to the Thr residue of ring A leads to a higher modification efficiency (Table 2, Figure 6). The expression yield of construct *m* is relatively high compared to all other tested ring A mutants (**S7**). It is possible that for this construct, a ring is formed from Cys3 to Thr4, as Glu-3 is in relative position −5 to these residues, which would meet the prerequisites for ring A formation.^17^ While it is possible that ring A is installed from Cys3 to Thr4, this would prevent the formation of ring B.

**Figure 7:**
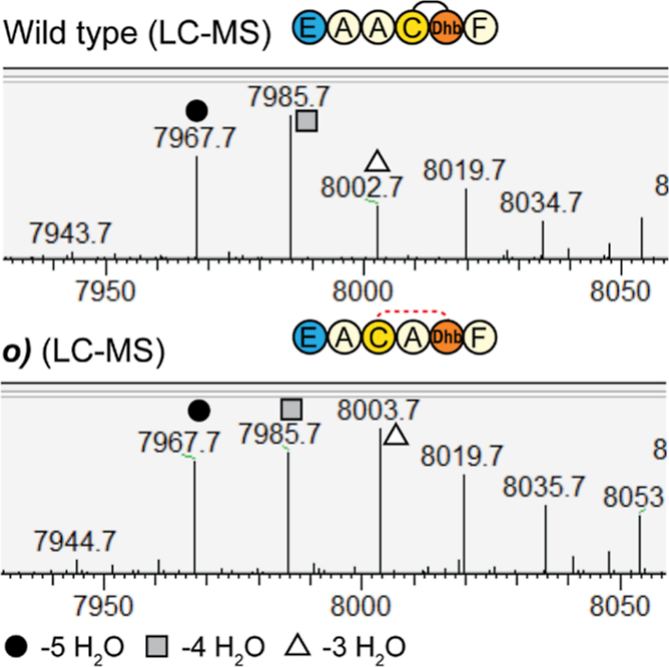
LC–MS analysis of ring A mutant *o*, which has an increased ring A size. LC–MS analysis of ring A mutant *o* shows a dehydration pattern that is similar, yet with a lower modification efficiency, to the wildtype mersacidin (**S5**). To determine if all rings can still be formed, a NEM-free cysteine assay was performed (Figure 8).

**Figure 8:**
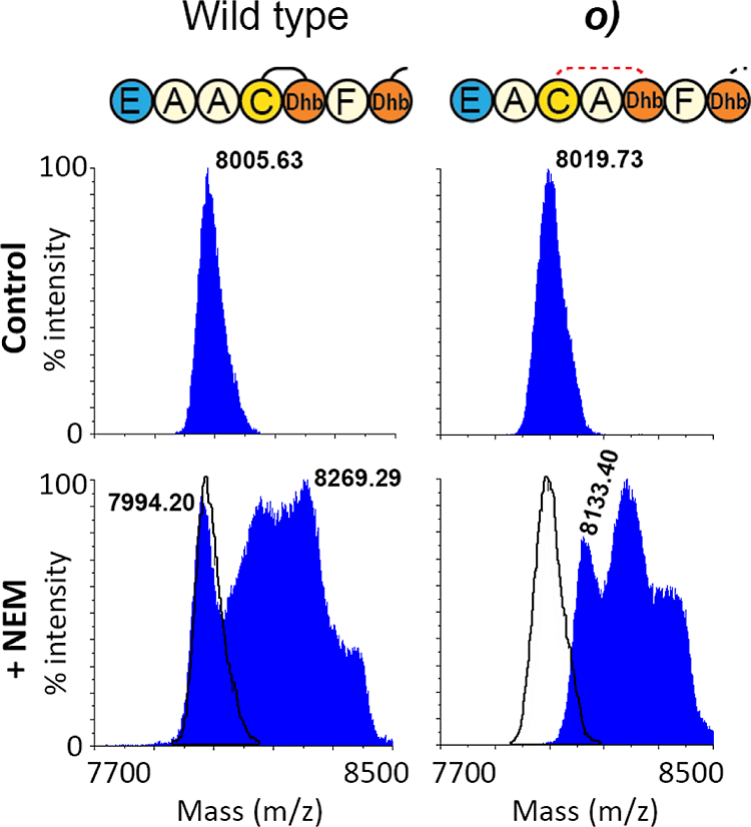
MALDI-TOF analysis of the NEM-free cysteine assay on ring A mutant *o*, which has an increased ring A size. Because construct *o* was shown to be fully dehydrated in the LC–MS analysis, a NEM-free cysteine assay was performed. As the differently dehydrated species show up as a centroid peak in MALDI-TOF analysis of products of this mass, the masses were confirmed by LC–MS prior to the free cysteine assay (**S4**). Any free cysteines should result in a shift of 125 Da. While the observed mass of the wildtype control partially undergoes mass shifts that indicate free cysteines, about 25% of the product does not shift and is thus fully modified. In contrast, all of the products of construct *o* shift after the free cysteine assay, meaning that in none of the products, all rings are formed and that it is thus not possible to increase the size of ring A by inserting an alanine, even when restoring the distance from Asp-5 to the Thr of ring A to six residues.

Finally, a construct was tested that increased the ring size of ring A by the insertion of an Ala residue while keeping the original direction intact (Table 2*o*, Figure 7). While it has previously been shown that the size of ring A cannot be increased,^23^ the recently elucidated importance of the GDMEAA sequence and its distance to the core peptide suggests that it might be possible to take a different approach. By increasing the ring size, the distance of Asp-5 to the Thr of ring A is increased by one residue, decreasing dehydration efficiency. Next to having an increased ring size, construct *o* has an Ala removed from the GDMEAA sequence to restore the distance of Asp-5 to the Thr residue of Ring A to its original length. LC–MS analysis of construct *o* revealed that it could be fully dehydrated (Figure 7) (**S5**), but the subsequent free cysteine assay showed that it cannot be fully cyclized (Figure 8). It is not certain why all dehydrations could occur in construct *o*, but in none of the other constructs, especially construct *l*. A clear difference in construct *o* is that its Cys1 residue has the largest distance to ring B, but other factors such as influences on the secondary structure of the peptide might well be involved.

It is clear that the formation of ring A and possibly ring B have multiple dependencies on specific surrounding amino acid sequences for correct modification to occur. In the formation of ring A of mersacidin, the distance from Asp-5 and Glu-3 to Thr2 plays an important role in the dehydration efficiency. For ring A formation to occur, it is crucial that residue Cys1 is directly upstream of Thr2. When compensating one parameter by, for example, the addition or removal of an extra amino acid to facilitate dehydration, ring formation no longer occurs, and vice versa. Additionally, the position of the residues of ring A impact the maturation of the rest of the peptide.

Just like the truncated variants, all of the ring A mutants were scanned for antimicrobial activity after leader peptide removal with AprE-His.^16^ No activity could be detected for these variants, which was expected since none of the mutants was fully modified (**S8**).

Many more single amino acid mutants can be conceived that could shed more light on mersacidin maturation, such as mutants of Phe3 of the core peptide. Previously, a mutational analysis of mersacidin has been reported, in which 12 mutants of Phe3 were tested.^23^ Some of these mutants were better expressed than others, but no obvious pattern can be identified between expression levels and amino acid traits such as hydrophobicity or size.

While it would be interesting to test such mutants in a simplified environment, such as testing a Phe3 mutant of construct *f*, results from such experiments would not allow for conclusive statements on the order of mersacidin’s ring formation. As is shown here, ring A can be formed without the presence of ring B in construct *f*. However, when the amino acid sequence for ring B is also present, like in construct *a*, the first ring is no longer formed. When Ring C and D are also present, like in wildtype mersacidin, ring *a* can be formed again. Ring B is thus not crucial for the formation of ring A, but from these results, it cannot be derived whether A is formed before ring B in wildtype mersacidin, merely that it is physically possible. Likewise, mutants of Phe3 or any other single amino acids could interfere with the formation of secondary structures that affect the formation of any ring of mersacidin. Effectively, single amino acid mutants cannot be the sole strategy for determining the order of ring formation in mersacidin.

### Perspectives

Although the flexibility of ring A formation could be further explored to some extent, it is becoming increasingly evident that the GDMEAA sequence and the subsequent ring A structure function as a cassette with little flexibility. And so, while the amino acid sequence downstream of ring A can probably still be improved, the original ring A should be applied as a nonchanging module. While different amino acid substitutions have already been performed on the residues downstream of ring A,^23^ the effect of these mutations was tested against the maturation efficiency of the whole mersacidin molecule instead of just ring A. Therefore, additional mutation analysis is needed to optimize ring A formation. Additionally, the expression protocols that are optimized for mersacidin production might not be optimal for ring A expression and could therefore potentially be improved upon. Since expression of wildtype mersacidin in *Bacillus*(1) species leads to higher yields than expression in *E. coli*,^13^ Mini*Bacillus* PG10^24^ could be a suitable production strain for the ring A module. In PG10, the native transporter MrsT may be employed to further increase expression yield. Since MrsT has been shown to recognize and cleave part of the leader peptide even in the absence of the core peptide,^16^ it would likely be able to export the ring A module. While in such a system the N-terminal His-tag would be cleaved upon export, its purification from the supernatant, rather than the intracellular fraction, can be done through other methods.

**Figure 9:**
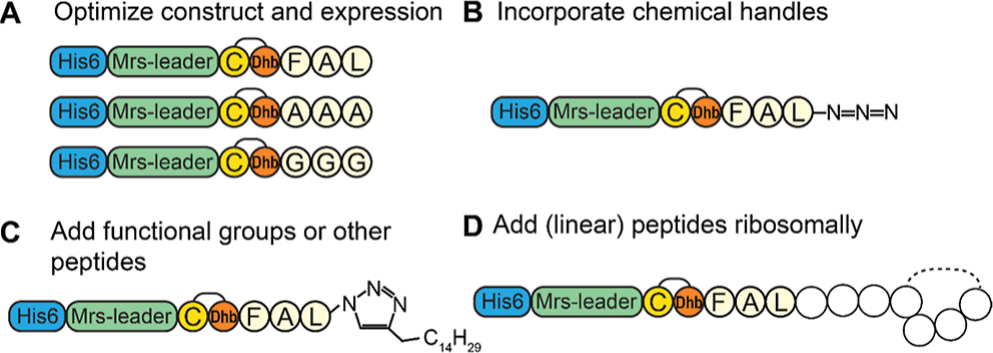
Suggestions for further optimization and application of the uniquely small ring A of mersacidin in RiPP engineering. (A) Efficiency of ring A formation can potentially be improved by optimizing residues downstream of the ring in combination with expression optimization. (B) Chemical handles can be installed, for example, through chemical addition to the C-terminal carboxyl group^20,27^ or the incorporation of noncanonical amino acids. (C) After chemical handles have been installed, functional groups such as fatty acid chains can be added to the ring A construct to obtain the intended functionality.^20^ (D) Linear peptides can be added to the minimal ring a construct in a ribosomal way to obtain lanthionine-stabilized linear peptides.

The minimal ring A core peptide CTFAL can be used as a very small lanthipeptide module for chemical modifications. These can be installed through the incorporation of noncanonical amino acids with chemical handles,^6,22,25,26^ to which functional moieties can be added through chemical addition in vitro (Figure 9). Alternatively, in vitro chemical additions can be performed on the negatively charged C-terminus.^20,27^ The approaches for the chemical modification of lanthipeptides have already been reported in the literature,^6^ and only the compatibility of MrsM with noncanonical amino acids can potentially cause difficulties. However, since the noncanonical amino acids can be positioned downstream of ring A, such difficulties can theoretically be prevented.

Since MrsM can dehydrate serine residues without forming a ring, and MrsM has been found in this study to form a dehydrobutyrine from residue Thr4, which cannot form a ring in construct *b*, *d*, and *e*, another option for in vitro modification is available. Reported modifications of dehydrated residues through Diels–Alders^28^ addition and Cu II—catalyzed β-borylation^29^ offer more design options in the creation of lanthionine-stabilized new molecules.

An option allowing for even more freedom of the design would be the creation of a truncated mersacidin construct that contains ring A and the recognition sequence of MrsD, allowing the peptide’s C-terminus to be decarboxylated. The introduction of negatively charged amino acids into this peptide would then allow for the direction of chemical additions to the carboxyl group of Asp or Glu residues. Substitution of Glu17, which is probably in the MrsD recognition sequence, has already been shown to not prevent decarboxylation of the C-terminus,^23^ and it can thus be removed to facilitate the insertion of negative charges at different sites. While this approach requires more preparation work and characterization to be done, it can allow for the creation of even more exciting new molecules.

## Conclusions

Here, the smallest construct containing ring A of mersacidin has been determined to be the pentapeptide CTFAL. This construct has a good yield, decent modification efficiency, naturally low heterogeneity and it can be easily purified by HPLC. Additionally, it offers opportunities for further optimization and diversification. The formation of ring A itself was found to be quite stringent, and a reversal or expansion of this ring does not seem to be possible. Taking all this into account, the application of ring A of mersacidin in the form of the module CTFAL or its derivatives offers good opportunities for the creation of new and useful molecules through RiPP engineering using mersacidin modification enzymes and elements.

## Materials and Methods

### Bacterial Strains and Growth Conditions

For all cloning purposes, *E. coli* TOP10 was used, and *E. coli* BL21(DE3) was used for all expression purposes. *M. flavus* was used as the indicator strain in antimicrobial activity tests. All bacterial strains were grown in LB medium (Foremedium) at 225 rpm or on LB agar plates (Foremedium) at 37 °C unless stated otherwise. When growing *E. coli* strains with the plasmids pACYC or pBAD, growth media were supplemented with 15 μg/mL chloramphenicol or 100 μg/mL ampicillin, respectively.

### Molecular Cloning

All molecular cloning was done according to well-established protocols^30^ supplemented with manufacturer directions. All constructs created in this study (**S1**) were derived through mutagenic round PCR from pACYC His-MrsA + MrsM or pACYC His-MrsA in the case of negative control plasmids.^13^ Mutagenic primers were designed to introduce desired mutations and compatible Eco31I overhangs and ordered from Biolegio (Nijmegen, The Netherlands) (**S2**). PCR reactions to obtain the linear fragments were done using Phusion polymerase (Thermo Scientific), and products were purified using a NucleoSpin Gel and PCR clean-up kit (Macherey-Nagel). The cleaned-up products were digested using FastDigest Eco31I and DpnI restriction enzymes (Thermo Scientific), after which they were subjected to a second clean-up step. Next, the Eco31I-digested linear DNA was self-ligated using T4 ligase (Thermo Scientific), after which E. coli TOP10 was transformed with the resulting ligated plasmid DNA. Several colonies from the transformation were picked up and grown overnight, after which the overnight cultures were used to make glycerol stocks and to isolate the plasmid DNA using a NucleoSpin Plasmid EasyPure kit (Macherey-Nagel). The purified plasmid DNA was sent to Macrogen Europe (Amsterdam, The Netherlands) for sequencing. Plasmid DNA from positive clones was used to transform E. coli BL21(DE3) for expression purposes.

### Peptide and Protein Expression

For the expression of truncated mersacidin constructs and ring A variants in combination with MrsM, fresh transformants of *E. coli* BL21(DE3) were created using the relevant pACYC plasmid DNA, with the addition of pBAD MrsD in the case of the nontruncated ring A mutants. Several colonies were picked up for each expression and grown overnight. Then, they were diluted 100× in 300 mL of fresh medium and grown for 2.5 h. Next, the cultures were cooled to 16 °C in an ice water bath, after which they were induced with 1 mM IPTG (pACYC) and 0.2% arabinose (pBAD). After induction, the cultures were grown at 16 °C for 29 h, after which the cells were harvested.

Expression of the truncated mersacidin constructs without MrsM, which functioned as negative controls in the free cysteine assays, was done in a similar way with the following exceptions. To prevent degradation, the cultures were not cooled, both were induced at 37 °C, and then grown for 4 h at 37 °C before harvesting the cells.

Expression and purification of the protease AprE-His, which was used in the antimicrobial activity tests, was done as previously described.^16^

### Peptide Purification

All expressed peptides contain a His-tag, and they were initially purified through Ni-NTA chromatography. All buffers contained 20 mM H_2_NaPO_4_ (Merck) and 0.5 M NaCl (VWR), and they were set at pH 7.4. The binding, wash, and elution buffer contained 20, 50, and 500 mM imidazole (Merck), respectively. After spinning down the expression cultures, they were washed using 25 mL of binding buffer (bb) and then resuspended in 10 mL bb. Sonication was used to lyse the cells, after which the insoluble fragments were removed by centrifugation. A Ni-NTA chromatography column was prepared by pipetting 0.9 mL of Ni-NTA agarose slurry (Qiagen) into an empty column, resulting in a column volume (CV) of ca. 0.45 mL. After calibrating the column with 3 CV of bb, the cell lysate supernatant was loaded onto it. The column was washed using 10 CV of bb and then washed again using 10 CV of wash buffer. Finally, the peptide was eluted from the column using 4 CV of elution buffer.

After Ni-NTA chromatography, all samples were further purified by reversed-phase chromatography using an open C-18 column. The samples were prepared by acidifying them by adding 0.5% trifluoroacetic acid (TFA) (Sigma) solution until pH < 4.0. The column was prepared by adding 0.25 g of 55–105 μm C18 resin (Waters) to an empty column, resulting in a CV of ca. 1 mL. After wetting the column with 2 CV of acetonitrile (ACN) (VWR) + 0.1% TFA, it was calibrated with Milli-Q + 0.1% TFA, and the sample was loaded onto the column. Next, the column was washed with 10 CV Milli-Q + 0.1% TFA, after which it was washed again with 5 CV 20% ACN + 0.1% TFA. Finally, the sample was eluted in 4 mL of 50% ACN + 0.1% TFA. The elution samples were freeze-dried and stored at −20 °C.

To prepare the samples for HPLC purification, they were dissolved in 150 μL of Milli-Q water. 125 μL of this solution was added to 175 μL of Milli-Q water, setting the total volume to 300 μL. This solution was then filtered (0.2 μm) and injected following the described protocol (**S3**).

### Free Cysteine Assays and Mass Spectrometry

LC–MS and MALDI-TOF mass spectrometry were performed as previously described.^31^ TCEP reduction of samples was done by adding 1 mM TCEP HCl to 10 μL of the dissolved peptide and incubating for 5 min at room temperature. The IAA-free cysteine assay of TCEP-reduced samples was done by adjusting the pH of the sample to 7 by adding 100 mM ammonium bicarbonate buffer and then adding 55 mM IAA. The sample was then incubated for 30 min at room temperature and used to perform LC–MS analysis. The *N*-ethylmaleimide (NEM)-free cysteine assay was performed as described previously.^17^

### Tricine SDS-page

Tricine SDS-page gels were prepared as described previously.^32^ For each sample, 12 μL of Ni- NTA chromatography elution sample was mixed with 4 μL of 5× loading buffer [550 mM dithiothreitol (Sigma-Aldrich), 250 mM Tris–HCl (Boom), 50% glycerol (Boom), 10% sodium dodecyl sulfate (Sigma-Aldrich), 0.5% Coomassie Blue R-250 (Bio-Rad), pH 7.0]. The gels were run using the described protocol with a prestained marker (PageRuler, Thermo Scientific).

### Antimicrobial Activity Tests

For all antimicrobial activity tests, the samples were digested using AprE-His. For the mutants, 8 μL of freeze-dried peptide, dissolved in 150 μL of Milli-Q water, was added, and 2 μL of the peptide was used for the wildtype control. To each sample, 1 μL of AprE-His was added, after which the volume was set to 10 μL by adding Milli-Q water. The digestions were incubated at 37 °C for 1 h. To prepare antimicrobial activity plates, a 50–50 mixture of LB broth and LB agar was prepared. When hand warm, a fresh overnight culture of *M. flavus* was diluted 1000 times in this mixture. 12 mL of the resulting indicator mixture was used to create each activity plate. 9 μL of each digested peptide and the positive control of 25 ng/μL nisin were spotted on the plate. The plates were incubated overnight at 30 °C.

## Supporting Information Available

Amino acid sequences of all constructs; list of primers used in this study; HPLC protocol truncated mersacidin variants; HPLC spectra, LC–MS and free cysteine assays of truncated mersacidin mutants; LC–MS Ring A mutants; mass table of mersacidin variants; tricine SDS-page of all mutants; activity tests all mutants (PDF)

## References

[ref1] Bierbaum G.; Brötz H.; Koller K.-P.; Sahl H.-G. Cloning, Sequencing and Production of the Lantibiotic Mersacidin. FEMS Microbiol. Lett. 1995, 127, 121–126. 10.1111/j.1574-6968.1995.tb07460.x. 7737474

[ref2] Kruszewska D.; Sahl H.-G. G.; Bierbaum G.; Pag U.; Hynes S. O.; Ljungh Å. Mersacidin Eradicates Methicillin-Resistant Staphylococcus Aureus (MRSA) in a Mouse Rhinitis Model. J. Antimicrob. Chemother. 2004, 54, 648–653. 10.1093/jac/dkh387. 15282239

[ref3] Sass P.; Jansen A.; Szekat C.; Sass V.; Sahl H. G.; Bierbaum G. The Lantibiotic Mersacidin Is a Strong Inducer of the Cell Wall Stress Response of Staphylococcus Aureus. BMC Microbiol. 2008, 8, 1–11. 10.1186/1471-2180-8-186. 18947397 PMC2592248

[ref4] Arnison P. G.; Bibb M. J.; Bierbaum G.; Bowers A. A.; Bugni T. S.; Bulaj G.; Camarero J. A.; Campopiano D. J.; Challis G. L.; Clardy J.; Cotter P. D.; Craik D. J.; Dawson M.; Dittmann E.; Donadio S.; Dorrestein P. C.; Entian K.-D.; Fischbach M. A.; Garavelli J. S.; Göransson U.; Gruber C. W.; Haft D. H.; Hemscheidt T. K.; Hertweck C.; Hill C.; Horswill A. R.; Jaspars M.; Kelly W. L.; Klinman J. P.; Kuipers O. P.; Link A. J.; Liu W.; Marahiel M. A.; Mitchell D. A.; Moll G. N.; Moore B. S.; Müller R.; Nair S. K.; Nes I. F.; Norris G. E.; Olivera B. M.; Onaka H.; Patchett M. L.; Piel J.; Reaney M. J. T.; Rebuffat S.; Ross R. P.; Sahl H.-G.; Schmidt E. W.; Selsted M. E.; Severinov K.; Shen B.; Sivonen K.; Smith L.; Stein T.; Süssmuth R. D.; Tagg J. R.; Tang G.-L.; Truman A. W.; Vederas J. C.; Walsh C. T.; Walton J. D.; Wenzel S. C.; Willey J. M.; van der Donk W. A. Ribosomally Synthesized and Post-Translationally Modified Peptide Natural Products: Overview and Recommendations for a Universal Nomenclature. Nat. Prod. Rep. 2013, 30, 108–160. 10.1039/C2NP20085F. 23165928 PMC3954855

[ref5] Field D.; Cotter P. D.; Hill C.; Ross R. P. Bioengineering Lantibiotics for Therapeutic Success. Front. Microbiol. 2015, 6, 1363 10.3389/fmicb.2015.01363. 26640466 PMC4662063

[ref6] Montalbán-López M.; Scott T. A.; Ramesh S.; Rahman I. R.; van Heel A. J.; Viel J. H.; Bandarian V.; Dittmann E.; Genilloud O.; Goto Y.; Grande Burgos M. J.; Hill C.; Kim S.; Koehnke J.; Latham J. A.; Link A. J.; Martínez B.; Nair S. K.; Nicolet Y.; Rebuffat S.; Sahl H.-G.; Sareen D.; Schmidt E. W.; Schmitt L.; Severinov K.; Süssmuth R. D.; Truman A. W.; Wang H.; Weng J.-K.; van Wezel G. P.; Zhang Q.; Zhong J.; Piel J.; Mitchell D. A.; Kuipers O. P.; van der Donk W. A. New Developments in RiPP Discovery, Enzymology and Engineering. Nat. Prod. Rep. 2021, 38, 130–239. 10.1039/D0NP00027B. 32935693 PMC7864896

[ref7] Repka L. M.; Chekan J. R.; Nair S. K.; van der Donk W. A. Mechanistic Understanding of Lanthipeptide Biosynthetic Enzymes. Chem. Rev. 2017, 117, 5457–5520. 10.1021/acs.chemrev.6b00591. 28135077 PMC5408752

[ref8] Sahl H.-G.; Bierbaum G. Lantibiotics: Biosynthesis and Biological Activities of Uniquely Modified Peptides from Gram-Positive Bacteria. Annu. Rev. Microbiol. 1998, 52, 41–79. 10.1146/annurev.micro.52.1.41. 9891793

[ref9] Suda S.; Westerbeek A.; O’Connor P. M.; Ross R. P.; Hill C.; Cotter P. D. Effect of Bioengineering Lacticin 3147 Lanthionine Bridges on Specific Activity and Resistance to Heat and Proteases. Chem. Biol. 2010, 17, 1151–1160. 10.1016/j.chembiol.2010.08.011. 21035738

[ref10] Guder A.; Schmitter T.; Wiedemann I.; Sahl H. G.; Bierbaum G. Role of the Single Regulator MrsR1 and the Two-Component System MrsR2/K2 in the Regulation of Mersacidin Production and Immunity. Appl. Environ. Microbiol. 2002, 68, 106–113. 10.1128/AEM.68.1.106. 11772616 PMC126572

[ref11] Schmitz S.; Hoffmann A.; Szekat C.; Rudd B.; Bierbaum G. The Lantibiotic Mersacidin Is an Autoinducing Peptide. Appl. Environ. Microbiol. 2006, 72, 7270–7277. 10.1128/AEM.00723-06. 16980420 PMC1636175

[ref12] Altena K.; Guder A.; Cramer C.; Bierbaum G. Biosynthesis of the Lantibiotic Mersacidin: Organization of a Type B Lantibiotic Gene Cluster. Appl. Environ. Microbiol. 2000, 66, 2565–2571. 10.1128/AEM.66.6.2565-2571.2000. 10831439 PMC110582

[ref13] Viel J. H.; Jaarsma A. H.; Kuipers O. P. Heterologous Expression of Mersacidin in Escherichia Coli Elucidates the Mode of Leader Processing. ACS Synth. Biol. 2021, 10, 600–608. 10.1021/acssynbio.0c00601. 33689311 PMC7985838

[ref14] Majer F.; Schmid D. G.; Altena K.; Bierbaum G.; Kupke T. The Flavoprotein MrsD Catalyzes the Oxidative Decarboxylation Reaction Involved in Formation of the Peptidoglycan Biosynthesis Inhibitor Mersacidin. J. Bacteriol. 2002, 184, 1234–1243. 10.1128/JB.184.5.1234-1243.2002. 11844751 PMC134850

[ref15] Blaesse M.; Kupke T.; Huber R.; Steinbacher S. Structure of MrsD, an FAD-Binding Protein of the HFCD Family. Acta Crystallogr., Sect. D: Biol. Crystallogr. 2003, 59, 1414–1421. 10.1107/S0907444903011831. 12876343

[ref16] Viel J. H.; van Tilburg A. Y.; Kuipers O. P. Characterization of Leader Processing Shows That Partially Processed Mersacidin Is Activated by AprE After Export. Front. Microbiol. 2021, 12, 12 10.3389/fmicb.2021.765659. PMC8581636 34777321

[ref17] Viel J. H.; Kuipers O. P. Mutational Studies of the Mersacidin Leader Reveal the Function of Its Unique Two-Step Leader Processing Mechanism. ACS Synth. Biol. 2022, 11, 1949–1957. 10.1021/acssynbio.2c00088. 35504017 PMC9127955

[ref18] Kuipers A.; Moll G. N.; Wagner E.; Franklin R. Efficacy of Lanthionine-Stabilized Angiotensin-(1-7) in Type I and Type II Diabetes Mouse Models. Peptides 2019, 112, 78–84. 10.1016/j.peptides.2018.10.015. 30529303

[ref19] Mentlein R. Proline Residues in the Maturation and Degradation of Peptide Hormones and Neuropeptides. FEBS Lett. 1988, 234, 251–256. 10.1016/0014-5793(88)80092-9. 3292288

[ref20] Zhao X.; Xu Y.; Viel J. H.; Kuipers O. P. Semisynthetic Macrocyclic Lipo-Lanthipeptides Display Antimicrobial Activity against Bacterial Pathogens. ACS Synth. Biol. 2021, 10, 1980–1991. 10.1021/acssynbio.1c00161. 34347446 PMC8383303

[ref21] Hein C. D.; Liu X.-M.; Wang D. Click Chemistry, a Powerful Tool for Pharmaceutical Sciences. Pharm. Res. 2008, 25, 2216–2230. 10.1007/s11095-008-9616-1. 18509602 PMC2562613

[ref22] Deng J.; Viel J. H.; Chen J.; Kuipers O. P. Synthesis and Characterization of Heterodimers and Fluorescent Nisin Species by Incorporation of Methionine Analogues and Subsequent Click Chemistry. ACS Synth. Biol. 2020, 9, 2525–2536. 10.1021/acssynbio.0c00308. 32786360 PMC7507115

[ref23] Appleyard A. N.; Choi S.; Read D. M.; Lightfoot A.; Boakes S.; Hoffmann A.; Chopra I.; Bierbaum G.; Rudd B. A. M.; Dawson M. J.; Cortes J. Dissecting Structural and Functional Diversity of the Lantibiotic Mersacidin. Chem. Biol. 2009, 16, 490–498. 10.1016/j.chembiol.2009.03.011. 19477413 PMC2706954

[ref24] van Tilburg A. Y.; van Heel A. J.; Stülke J.; de Kok N. A. W.; Rueff A.-S.; Kuipers O. P. MiniBacillus PG10 as a Convenient and Effective Production Host for Lantibiotics. ACS Synth. Biol. 2020, 9, 1833–1842. 10.1021/acssynbio.0c00194. 32551553 PMC7372594

[ref25] Zhou L.; Shao J.; Li Q.; van Heel A. J.; de Vries M. P.; Broos J.; Kuipers O. P. Incorporation of Tryptophan Analogues into the Lantibiotic Nisin. Amino Acids 2016, 48, 1309–1318. 10.1007/s00726-016-2186-3. 26872656 PMC4833812

[ref26] Bartholomae M.; Baumann T.; Nickling J. H.; Peterhoff D.; Wagner R.; Budisa N.; Kuipers O. P. Expanding the Genetic Code of Lactococcus Lactis and Escherichia Coli to Incorporate Non-Canonical Amino Acids for Production of Modified Lantibiotics. Front. Microbiol. 2018, 9, 1–11. 10.3389/fmicb.2018.00657. 29681891 PMC5897534

[ref27] Deng J.-J.; Viel J. H.; Kubyshkin V.; Budisa N.; Kuipers O. P. Conjugation of Synthetic Polyproline Moietes to Lipid II Binding Fragments of Nisin Yields Active and Stable Antimicrobials. Front. Microbiol. 2020, 11, 575334 10.3389/fmicb.2020.575334. 33329435 PMC7715017

[ref28] Vries R. H.; Viel J. H.; Oudshoorn R.; Kuipers O. P.; Roelfes G. Selective Modification of Ribosomally Synthesized and Post-Translationally Modified Peptides (RiPPs) through Diels-Alder Cycloadditions on Dehydroalanine Residues. Chem.—Eur. J. 2019, 25, 12698–12702. 10.1002/chem.201902907. 31361053 PMC6790694

[ref29] Vries R. H.; Viel J. H.; Kuipers O. P.; Roelfes G. Rapid and Selective Chemical Editing of Ribosomally Synthesized and Post-Translationally Modified Peptides (RiPPs) via Cu II -Catalyzed β-Borylation of Dehydroamino Acids. Angew. Chem., Int. Ed. 2021, 60, 3946–3950. 10.1002/anie.202011460. PMC7898795 33185967

[ref30] Sambrook J.; Russel D. W. Molecular Cloning: A Laboratory Manual, 4th ed.; Cold Spring Harbor Laboratory Press: New York, U.S.A., 2001.

[ref31] Zhao X.; Yin Z.; Breukink E.; Moll G. N.; Kuipers O. P. An Engineered Double Lipid II Binding Motifs-Containing Lantibiotic Displays Potent and Selective Antimicrobial Activity against *Enterococcus Faecium*. Antimicrob. Agents Chemother. 2020, 64, e02050-19 10.1128/AAC.02050-19. 32179527 PMC7269505

[ref32] Schägger H. Tricine-SDS-PAGE. Nat. Protoc. 2006, 1, 16–22. 10.1038/nprot.2006.4. 17406207

