# Supplementary material for: Modular Use of the Uniquely Small Ring A of Mersacidin Generates the Smallest Ribosomally Produced Lanthipeptide
Source: ACS Synth Biol. 2022 Sep 6;11(9):3078–87. doi: 10.1021/acssynbio.2c00343 (PMC9486960; doi:10.1021/acssynbio.2c00343)
Supplement: Supplementary file 1 — sb2c00343_si_001.pdf [file sb2c00343_si_001.pdf]

Supporting Information to:

Modular Use of the Uniquely Small Ring A of Mersacidin Generates the Smallest Ribosomally Produced Lanthipeptide

Jakob H. Viel, Oscar P. Kuipers*

Department of Molecular Genetics, University of Groningen, Nijenborgh 7, 9747 AG, Groningen, The Netherlands.

***Correspondence:**
Oscar P. Kuipers
o.p.kuipers@rug.nl

Table of contents

S1. Amino acid sequences of all constructs 3
S2. List of primers used in this study 4
S3. HPLC protocol truncated mersacidin variants 5
S4. HPLC spectra, LC-MS and free cysteine assays of truncated mersacidin mutants 6
S5. LC-MS Ring A mutants 13
S6. Mass table of mersacidin variants 16
S7. Tricine SDS-page of all mutants 17
S8. Activity tests all mutants 18
References 19

S1. Amino acid sequences of all constructs

	**Truncated constructs**
**Wildtype**	MHHHHHHGSQEAIIRSWKDPFSRENSTQNPAGNPFSELKEAQMDKLV GAGDMEAACTFTLPGGGGVCTLTSECIC
a	MHHHHHHGSQEAIIRSWKDPFSRENSTQNPAGNPFSELKEAQMDKLV GAGDMEAACTFTLPGGGGVCIL
b	MHHHHHHGSQEAIIRSWKDPFSRENSTQNPAGNPFSELKEAQMDKLV GAGDMEAACTFTLPGGGGVNIL
c	MHHHHHHGSQEAIIRSWKDPFSRENSTQNPAGNPFSELKEAQMDKLV GAGDMEAACTFTLPGGGGVAIL
d	MHHHHHHGSQEAIIRSWKDPFSRENSTQNPAGNPFSELKEAQMDKLV GAGDMEAACTFTLPGGG
e	MHHHHHHGSQEAIIRSWKDPFSRENSTQNPAGNPFSELKEAQMDKLV GAGDMEAACTFTL
f	MHHHHHHGSQEAIIRSWKDPFSRENSTQNPAGNPFSELKEAQMDKLV GAGDMEAACTFAL
g	MHHHHHHGSQEAIIRSWKDPFSRENSTQNPAGNPFSELKEAQMDKLV GAGDMEAACTF
	**Ring A mutants**
**Wildtype**	MHHHHHHGSQEAIIRSWKDPFSRENSTQNPAGNPFSELKEAQMDKLV GAGDMEAACTFTLPGGGGVCTLTSECIC
h	MHHHHHHGSQEAIIRSWKDPFSRENSTQNPAGNPFSELKEAQMDKLV GAGDMEAACNFTLPGGGGVCTLTSECIC
i	MHHHHHHGSQEAIIRSWKDPFSRENSTQNPAGNPFSELKEAQMDKLV GAGDMEAACAFTLPGGGGVCTLTSECIC
j	MHHHHHHGSQEAIIRSWKDPFSRENSTQNPAGNPFSELKEAQMDKLV GAGDMEATCFTLPGGGGVCTLTSECIC
k	MHHHHHHGSQEAIIRSWKDPFSRENSTQNPAGNPFSELKEAQMDKLV GAGDMEAATCFTLPGGGGVCTLTSECIC
l	MHHHHHHGSQEAIIRSWKDPFSRENSTQNPAGNPFSELKEAQMDKLV GAGDMEAAATCFTLPGGGGVCTLTSECIC
m	MHHHHHHGSQEAIIRSWKDPFSRENSTQNPAGNPFSELKEAQMDKLV GAGDMEAATACTLPGGGGVCTLTSECIC
n	MHHHHHHGSQEAIIRSWKDPFSRENSTQNPAGNPFSELKEAQMDKLV GAGDMEAATACFTLPGGGGVCTLTSECIC
o	MHHHHHHGSQEAIIRSWKDPFSRENSTQNPAGNPFSELKEAQMDKLV GAGDMEACATFTLPGGGGVCTLTSECIC

S2. List of primers used in this study

	Sequence	Template	Result
fw	GACGGGTCTCCTTCTATAATTTAAGCTTTCTTTGAACCCTG	pACYC His-MrsA + MrsM[1]	pACYC *a* + MrsM
rv	GACGGGTCTCCAGAATACAAACACCGCCGCCACCAGGC		
fw	GACGGGTCTCCATTCTATAATTTAAGCTTTCTTTGAACCCTG		pACYC *b* + MrsM
rv	GACGGGTCTCCGAATATTAACACCGCCGCCACCAGGCAATG		
fw	GACGGGTCTCCGCGGCGGTGTTAATATTCTATAATTTAAGCTTTCTTTGAACCCTGCAGC		pACYC *c* + MrsM
rv	GACGGGTCTCCCCGCCACCAGGCAAGGCAAAAGTACATGCTGCTTCCATGTCTC		
fw	GACGGGTCTCCTAATTTAAGCTTTCTTTGAACCCTGCAGCAAGC		pACYC *d* + MrsM
rv	GACGGGTCTCCATTAGCCGCCACCAGGCAATGTAAAAG		
fw	GACGGGTCTCCATTGTAATTTAAGCTTTCTTTGAACCCTG		pACYC *e* + MrsM
rv	GACGGGTCTCCCAATGTAAAAGTACATGCTGCTTC		
fw	GACGGGTCTCCCGTTGTAATTTAAGCTTTCTTTGAACCCTGCAG		pACYC *f* + MrsM
rv	GACGGGTCTCCAACGCAAAAGTACATGCTGCTTCCATGTC		
fw	GTCAGGTCTCCTTTAATTTAAGCTTTCTTTGAACCC		pACYC *g* + MrsM
rv	GACGGGTCTCGTAAAAAGTACATGCTGCTTCCATGTC		
fw	GACGGGTCTCGCTTTACATTGCCTGGTGGCGGC		pACYC *h* + MrsM
rv	GACGGGTCTCGAAAGTTACATGCTGCTTCCATGTCTCCCG		
fw	GACGGGTCTCGCTTTACATTGCCTGGTGGCGGC		pACYC *i* + MrsM
rv	GACGGGTCTCGAAAGGCACATGCTGCTTCCATGTCTCCCG		
fw	GACGGGTCTCCCTTGTTTTACATTGCCTGGTGGCGGCG		pACYC *j* + MrsM
rv	GACGGGTCTCCCAAGTTGCTTCCATGTCTCCCGCACCTAC		
fw	GACGGGTCTCGCTTGTTTTACATTGCCTGGTGGCGGCGGTG		pACYC *k* + MrsM
rv	GACGGGTCTCGCAAGTTGCTGCTTCCATGTCTCCCGCACCTAC		
fw	GACGGGTCTCCGACTTGTTTTACATTGCCTGGTGGCGGCG		pACYC *l* + MrsM
rv	GACGGGTCTCCAGTCGCTGCTGCTTCCATGTCTCCCGCAC		
fw	GACGGGTCTCGCGTGTACATTGCCTGGTGGCGGCGGTGTTT		pACYC *m* + MrsM
rv	GACGGGTCTCGCACGCAGTTGCTGCTTCCATGTCTCCCGCACCTAC		
fw	GACGGGTCTCGCGTGTTTTACATTGCCTGGTGGCGGCGGTG		pACYC *n* + MrsM
rv	GACGGGTCTCGCACGCAGTTGCTGCTTCCATGTCTCCCGCACCTAC		
fw	GACGGGTCTCCGTGCGACTTTTACATTGCCTGGTGGCGGC		pACYC *o* + MrsM
rv	GACGGGTCTCCGCACATGCTTCCATGTCTCCCGCACCTAC		

S3. HPLC protocol truncated mersacidin variants

Time [min]	Milli-Q + 0.1% TFA	Acetonitrile + 0.1 % TFA	Flow [ml/min]
**0.00**	95.0	5.0	1.00
**2.00**	95.0	5.0	1.00
**3.00**	75.0	25.0	1.00
**4.00**	75.0	25.0	1.00
**30.00**	59.0	41.0	1.00
**30.10**	5.0	95.0	1.00
**35.00**	5.0	95.0	1.00
**35.10**	95.0	5.0	1.00

S4. HPLC spectra, LC-MS and free cysteine assays of truncated mersacidin mutants

a)

HPLC spectrum

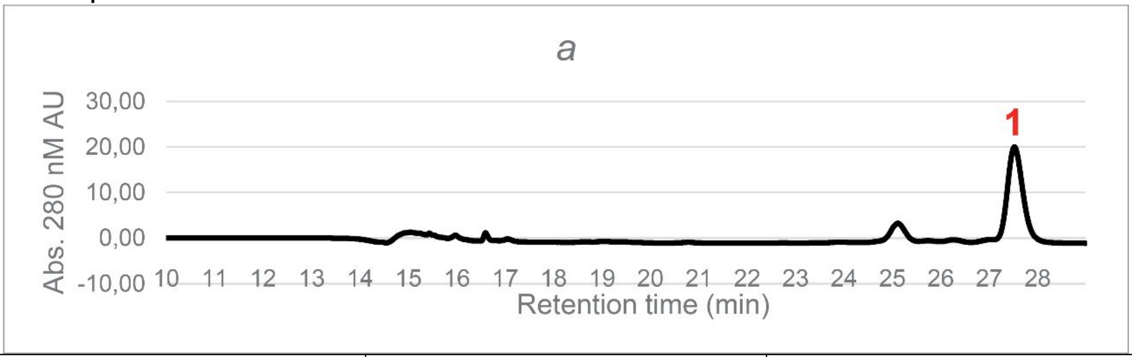

LC-MS Peak 1	Peak 1 + TCEP	Peak 1 + TCEP + IAA
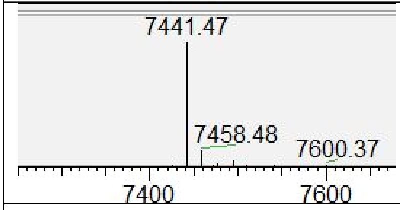	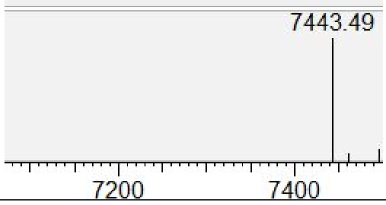	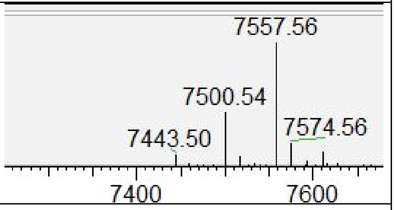

Peak 1 contains a disulfide bridge that can reduced by TCEP, leading to a 2 Da mass shift.

b)

HPLC spectrum

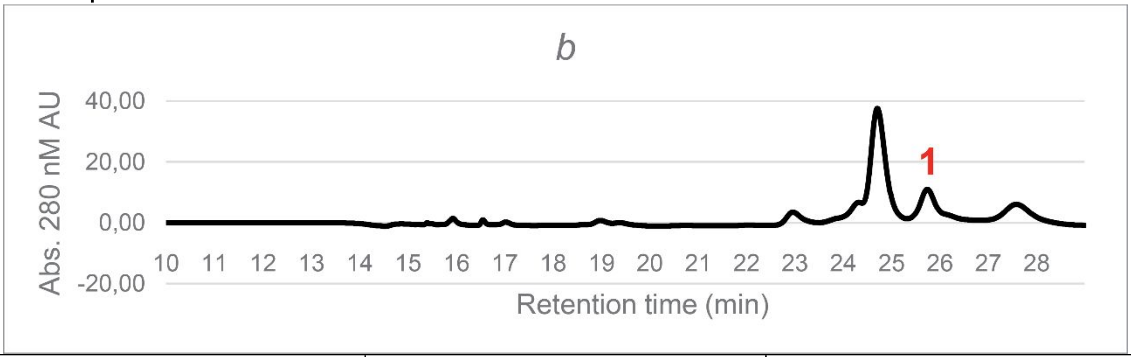

LC-MS Peak 1	Peak 1 + TCEP	Peak 1 + TCEP + IAA
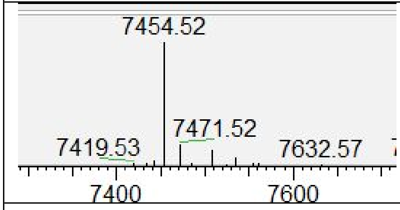	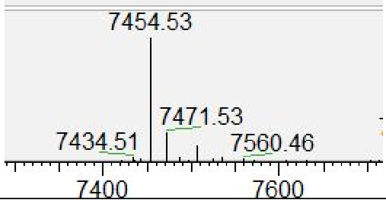	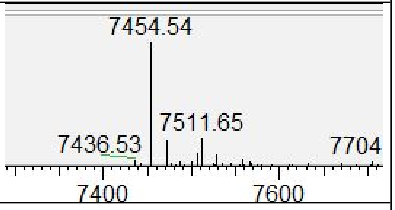

In peak 1, TCEP + IAA does not shift the expected mass for the dehydrated construct (**S7**), meaning that the ring was formed in this peak.

c)

HPLC spectrum

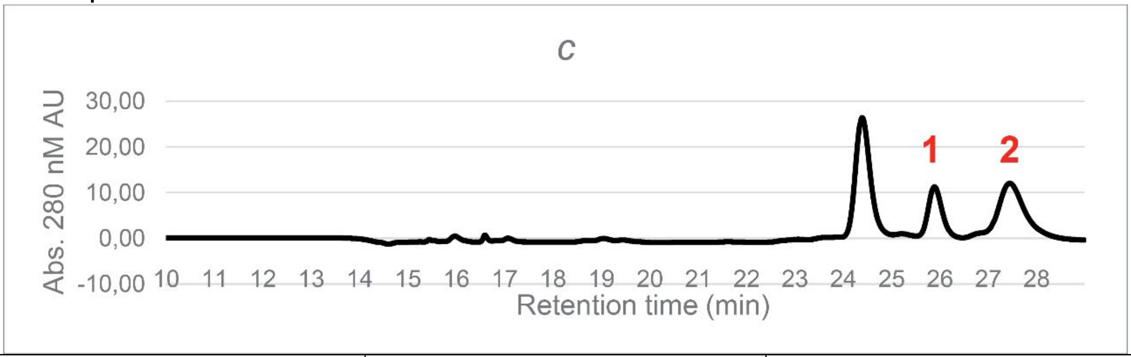

LC-MS Peak 1

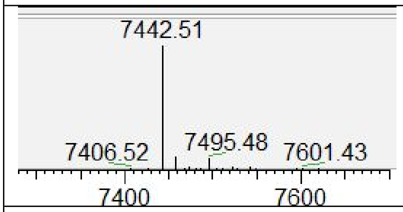

Peak 1 + TCEP

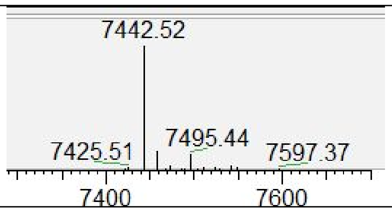

Peak 1 + TCEP + IAA

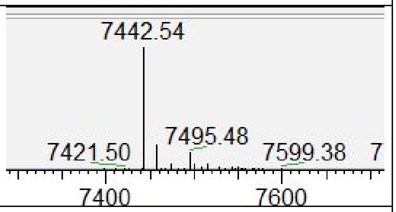

LC-MS Peak 2

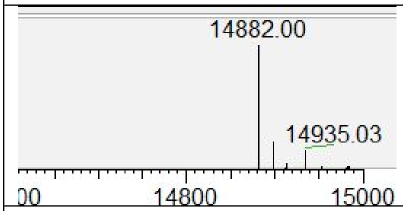

Peak 2 + TCEP

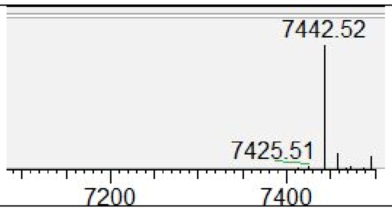

Peak 2 + TCEP + IAA

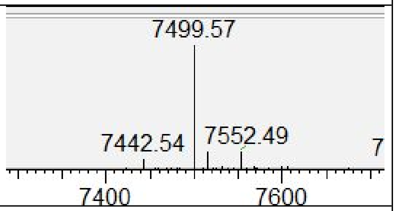

Peak 2 contains 1 x dehydrated disulfide bridge dimers lacking lanthionine rings (14882.00 Da), that is reduced to it's monomer (7442.52 Da) by TCEP. In peak 1, TCEP + IAA does not shift the expected mass for the dehydrated construct (**S7**), meaning that the ring was formed in this peak.

d)

HPLC spectrum

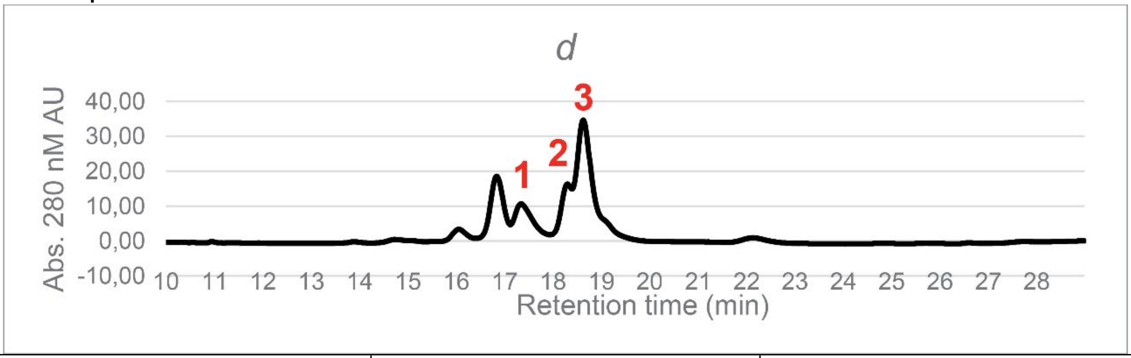

LC-MS Peak 1		Peak 1 + TCEP + IAA
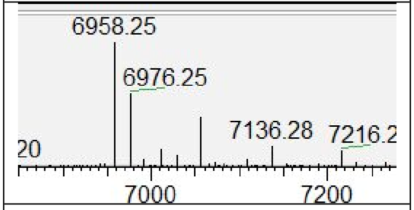		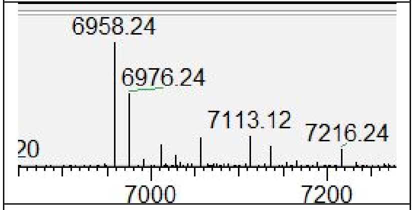
LC-MS Peak 2		Peak 2 + TCEP + IAA
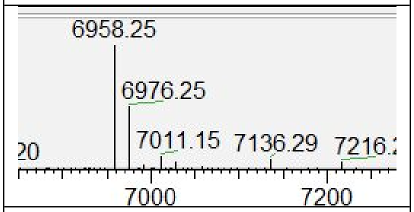		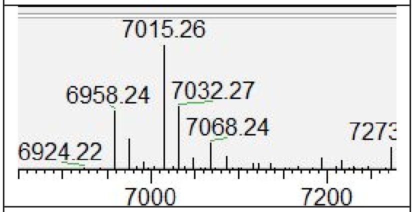
LC-MS Peak 3		Peak 3 + TCEP + IAA
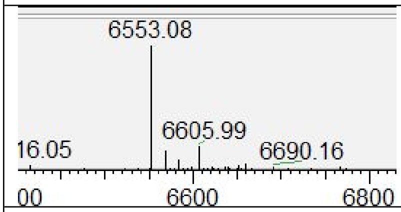		N/A

In peak 1, TCEP + IAA does not shift the expected mass for the dehydrated construct (**S7**), meaning that the ring was formed in this peak. In peak 2 most product shifted. In peak 3 the product had the wrong mass and was not further analyzed.

e)

HPLC spectrum

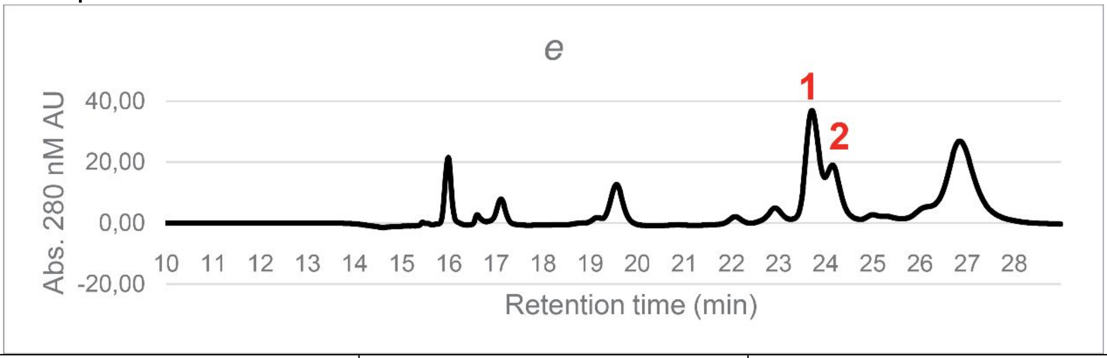

LC-MS Peak 1

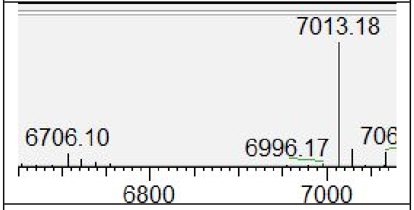

Peak 1 + TCEP + IAA

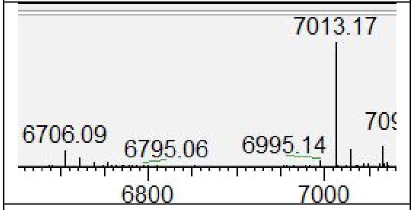

LC-MS Peak 2

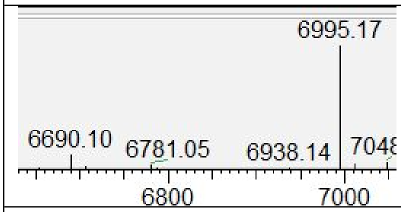

Peak 2 + TCEP + IAA

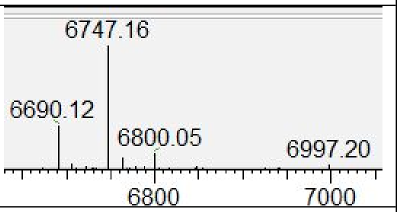

In peak 1, the main product does not have the expected mass, probably through an unkown adduct. In peak 2, TCEP + IAA shifts the expected mass for the dehydrated construct (**S7**), meaning that the ring was mostly not formed in this peak. For this construct atmost a bit of product is formed, and, since the peak of interest (2) overlaps with peak 1 containing a strange adduct, it would be hard to purify.

f)

HPLC spectrum

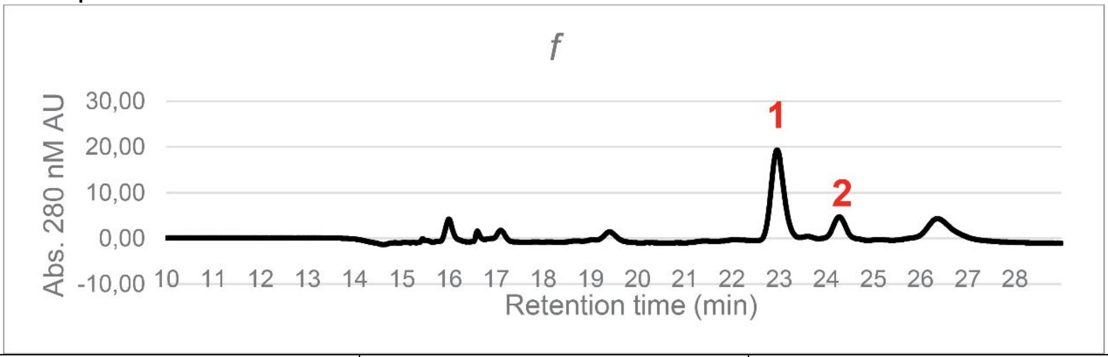

LC-MS Peak 1

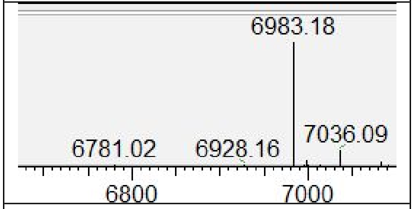

Peak 1 + TCEP + IAA

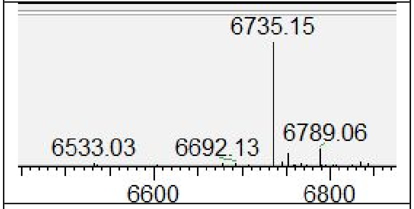

LC-MS Peak 2

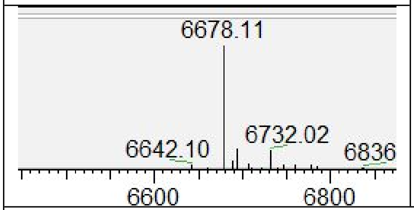

Peak 2 + TCEP + IAA

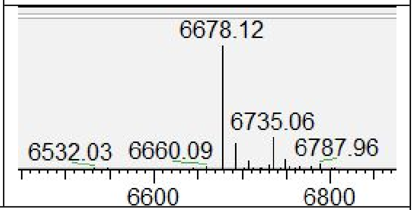

Peak 1, like peak 1 of construct *e,* has an unknown adduct of approximately 305 Da. The reduction and free cysteine essay, leads to removal of the adduct, but the resulting peak is the alkylated product, meaning the ring is not formed. Peak 2 has the correct mass, and the free cysteine essay causes almost no shift, meaning that the ring is formed in the majority of the product. Peak 2 is also easily separated from other products by HPLC, making it an attractive candidate future work.

g)

HPLC spectrum

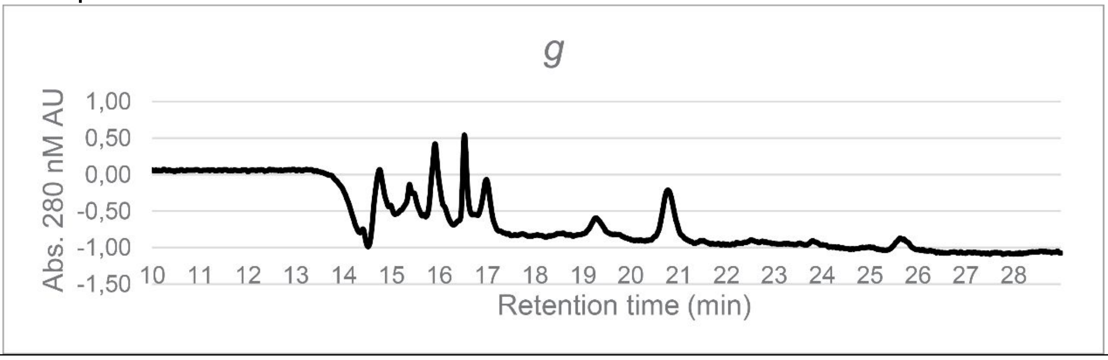

No major products could be purified for construct *g,* which is an indication that no dehydration can occur in this construct. Lack of dehydration and or ring formation leads to degradation during the long expression time for these constructs.

Control free cysteine assay unmodified construct *a*

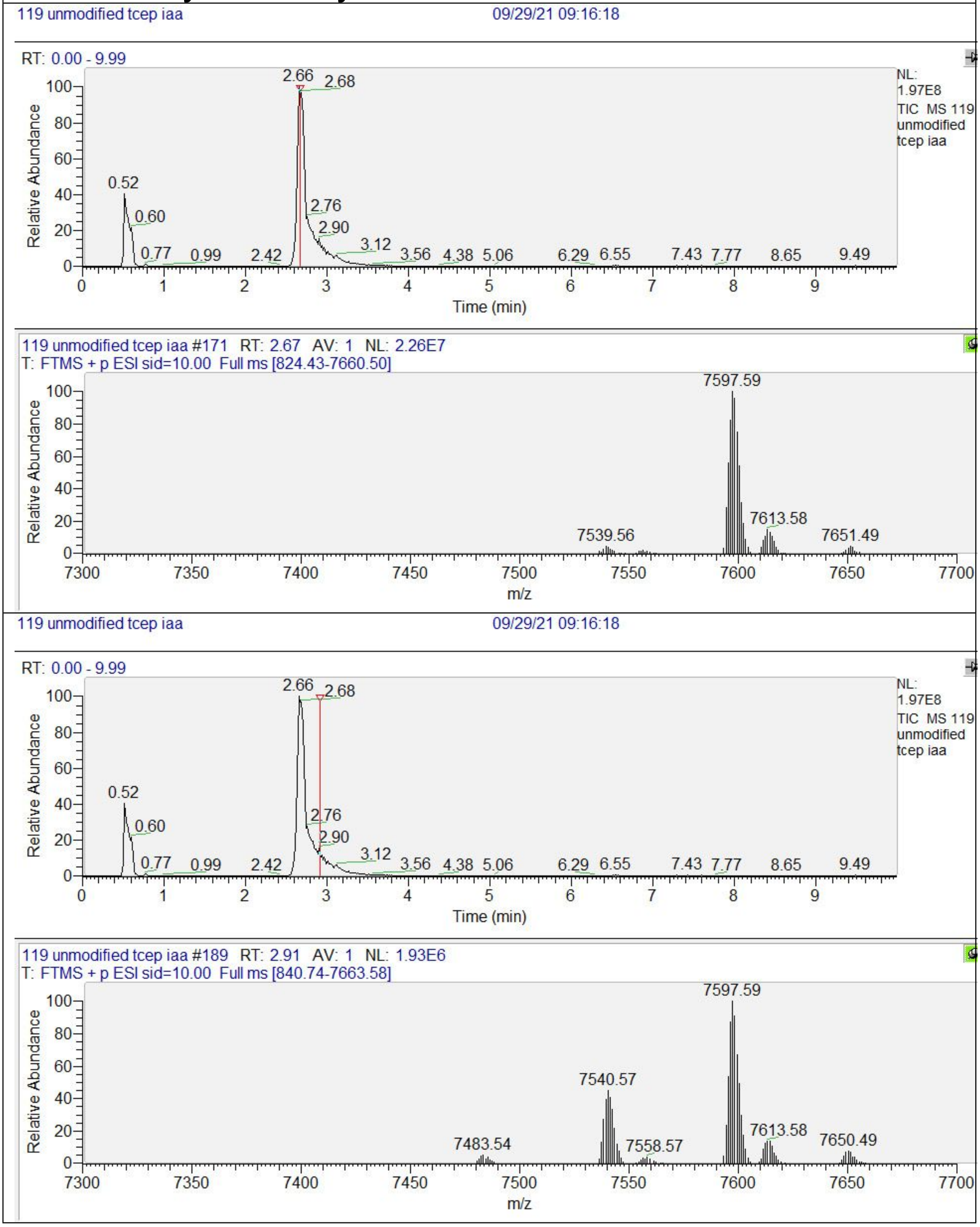

Because the results of the free cysteine essay of construct *a* didn't completely agree with the mass shift of the TCEP reduction (i.e., the fast majority rather than all of the peptide shifted), a free cysteine essay was done on construct *a* expressed without MrsM, that should thus always result in a shift resembling the alkylation of two cysteines. As can be seen in the top figure, the fast majority of the product has been alkylated twice (7597.59 Da, 7.598,45 Da theoretical), with trace amounts of once alkylated product (7539.56 Da, 7.541,38 Da theoretical). Small amounts of product with a higher retention time can be seen in the lower figure. Here the amount of once alkylated product is higher, and trace amounts of non-alkylated product are observed (7483.54 Da, 7484,305 Da theoretical). The control points out that trace amounts of product are not fully alkylated, which should be considered a

baseline of the free cysteine assays. And thus, from these experiments complete lack of ring formation cannot be confirmed. However, they are more than sufficient to compare ring formation efficiency between different mutants and confirm complete ring formation.

The same phenomenon can be seen in the control free cysteine essay of unmodified construct *c* (below). The cysteine residues in the majority of the product is completely alkylated (7521.91 Da, 7522.31 Da theoretical), with a trace amounts of non-alkylated peptide in the higher retention time products (7464.56 Da, 7465.24 Da theoretical (**S6**).

Control free cysteine assay unmodified construct *c*

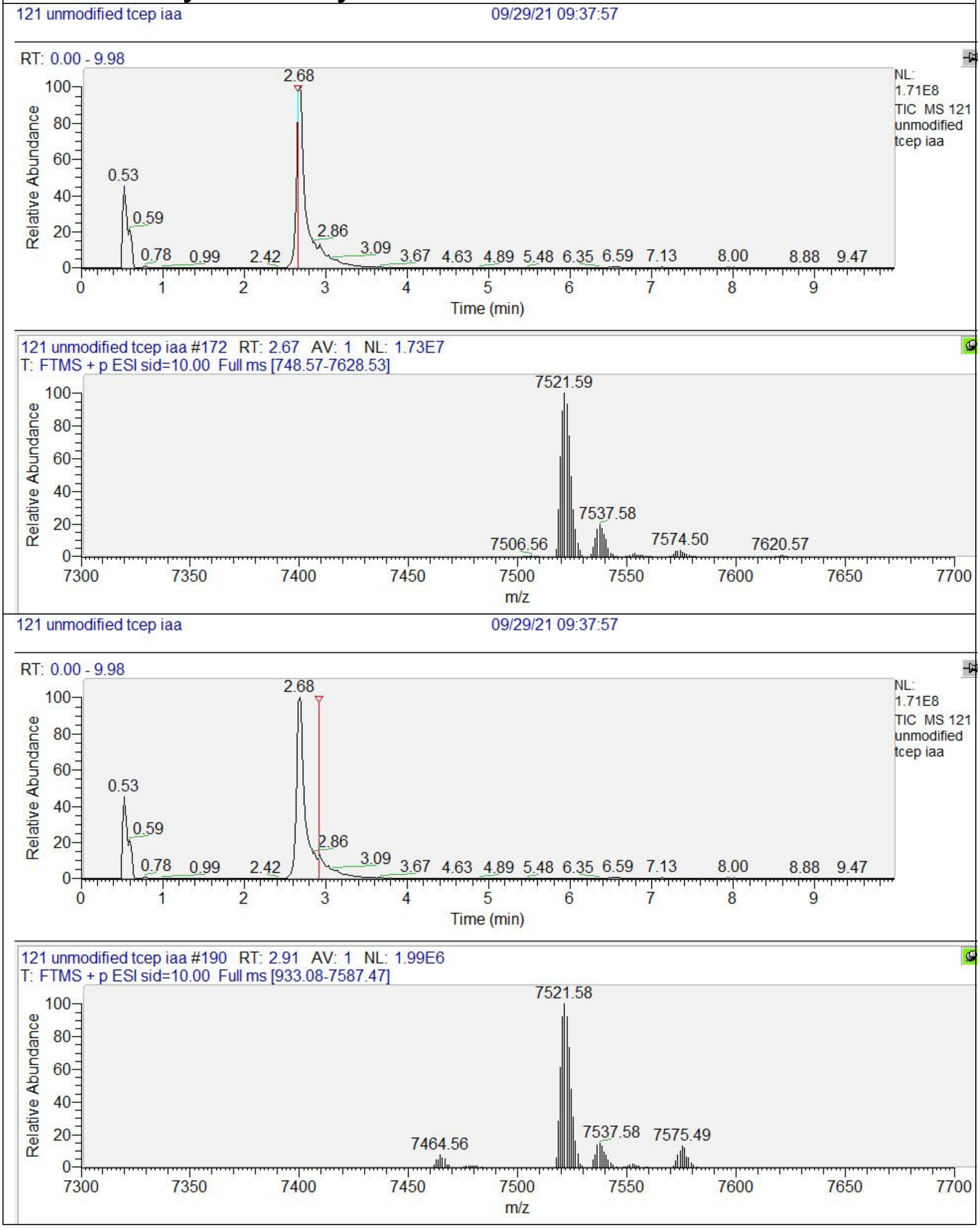

S5. LC-MS Ring A mutants

wildtype

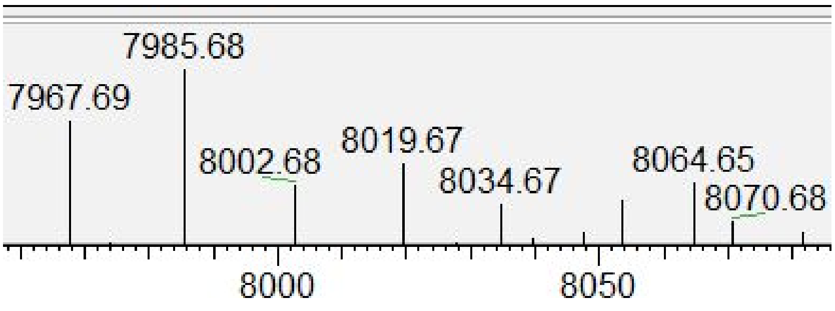

	mono-isotopic mass $-CO_2$
	8057,70
$-1H_2O$	8039,69
$-2H_2O$	8021,68
$-3H_2O$	8003,67
$-4H_2O$	7985,66
$-5H_2O$	7967,65

h

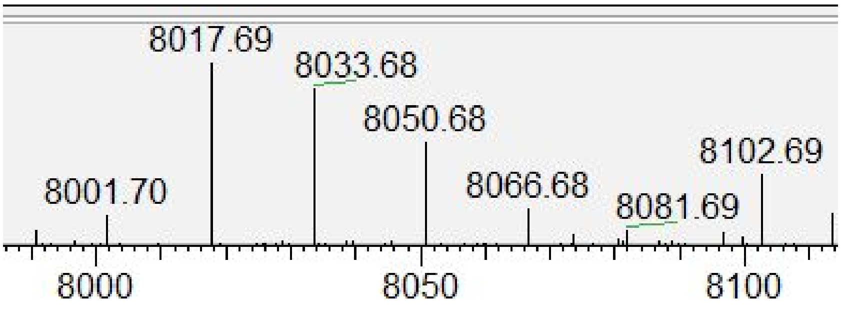

	mono-isotopic mass $-CO_2$
	8070,69
$-1H_2O$	8052,68
$-2H_2O$	8034,67
$-3H_2O$	8016,66
$-4H_2O$	7998,65
$-5H_2O$	7980,64

i

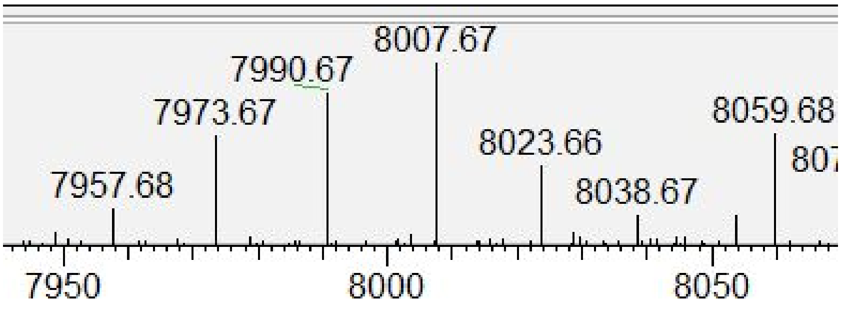

	mono-isotopic mass $-CO_2$
	8027,69
$-1H_2O$	8009,68
$-2H_2O$	7991,67
$-3H_2O$	7973,66
$-4H_2O$	7955,65
$-5H_2O$	7937,64

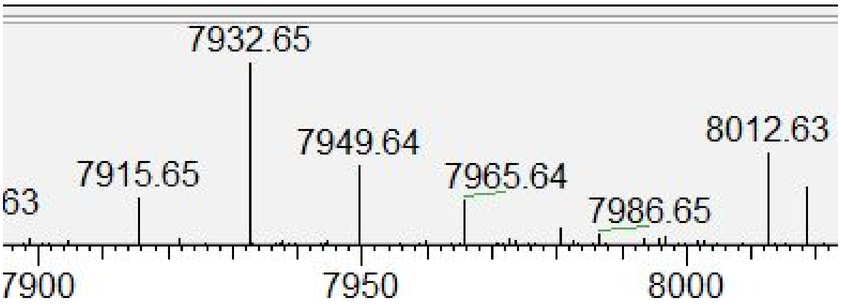

j	mono-isotopic mass $-CO_2$
	7986,66
$-1H_2O$	7968,65
$-2H_2O$	7950,64
$-3H_2O$	7932,63
$-4H_2O$	7914,62
$-5H_2O$	7896,61

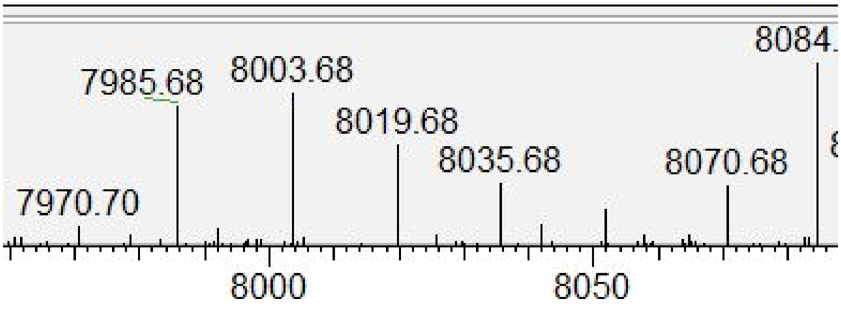

k	mono-isotopic mass $-CO_2$
	8057,70
$-1H_2O$	8039,69
$-2H_2O$	8021,68
$-3H_2O$	8003,67
$-4H_2O$	7985,66
$-5H_2O$	7967,65

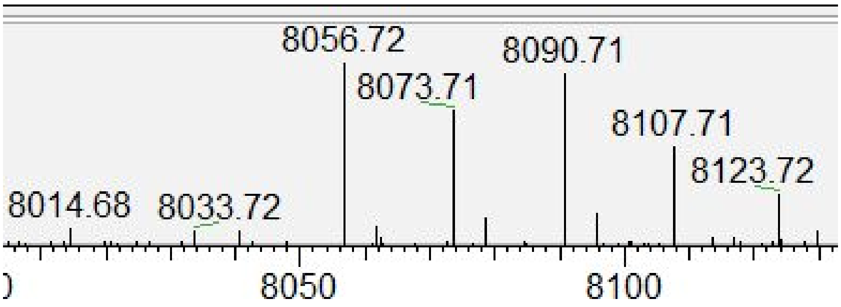

l	mono-isotopic mass $-CO_2$
	8128,74
$-1H_2O$	8110,72
$-2H_2O$	8092,71
$-3H_2O$	8074,70
$-4H_2O$	8056,69
$-5H_2O$	8038,68

m	mono-isotopic mass $-CO_2$	
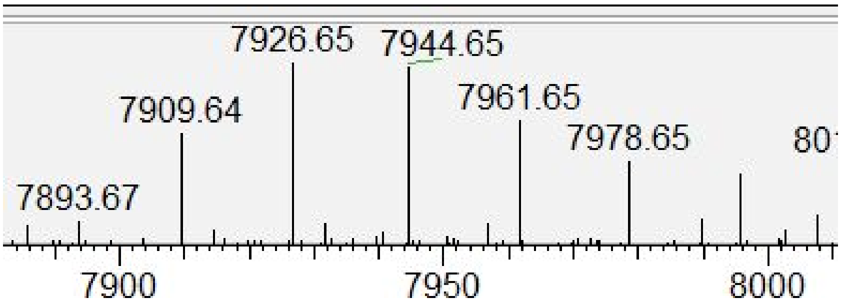		7981,67
	$-1H_2O$	7963,66
	$-2H_2O$	7945,65
	$-3H_2O$	7927,64
	$-4H_2O$	7909,62
	$-5H_2O$	7891,61

n	mono-isotopic mass $-CO_2$	
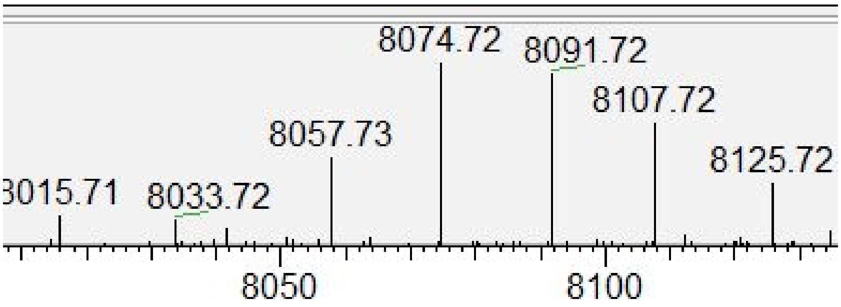		8128,74
	$-1H_2O$	8110,72
	$-2H_2O$	8092,71
	$-3H_2O$	8074,70
	$-4H_2O$	8056,69
	$-5H_2O$	8038,68

o	mono-isotopic mass $-CO_2$	
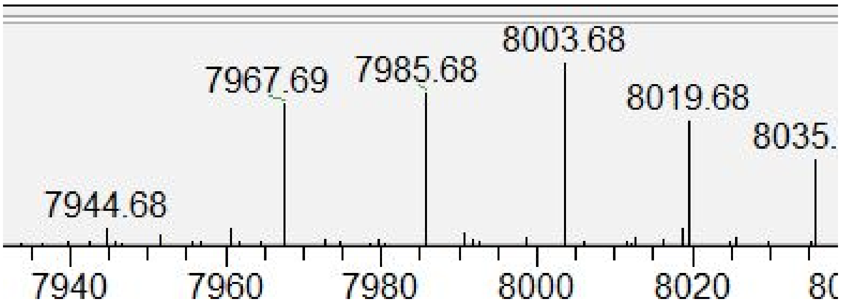		8057,70
	$-1H_2O$	8039,69
	$-2H_2O$	8021,68
	$-3H_2O$	8003,67
	$-4H_2O$	7985,66
	$-5H_2O$	7967,65

S6. Mass table of mersacidin variants

**Truncated mersacidin contructs (mono-isotopic)**							
Mono-isotopic masses (Da)							
			**+ IAA**		**+2 IAA**		
**Mutant**	Unmodified	- 1 x H_2O	- 2 x H_2O	- 1 x H_2O	- 2 x H_2O	- 1 x H_2O	- 2 x H_2O
a	7.479,52	7.461,51	7.443,49	7.518,58	7.500,56	7.575,65	7.557,64
b	7490,549	7.472,54	7.454,53	7.529,61	7.511,60	N/A	
c	7460,539	7.442,53	N/A	7.499,60	N/A		
d	6994,248	6.976,24	6.958,23	7.033,31	7.015,30		
e	6726,131	6.708,12	6.690,11	6.765,19	6.747,18		
f	6696,121	6.678,11	N/A	6.735,18	N/A		
g	6511,999	6.493,99	N/A	6.551,06	N/A		
**Ring A mutants**							
Mono-isotopic masses (Da)							
	Unmodified	$-CO_2$	**- 1** x H_2O	**- 2** x H_2O	**- 3** x H_2O	**- 4** x H_2O	**- 5** x H_2O
**wt**	8.103,70	8.057,70	8.039,69	8.021,68	8.003,67	7.985,66	7.967,65
h	8.116,70	8.070,69	8.052,68	8.034,67	8.016,66	7.998,65	7.980,64
i	8.073,69	8.027,69	8.009,68	7.991,67	7.973,66	7.955,65	7.937,64
j	8.032,67	7.986,66	7.968,65	7.950,64	7.932,63	7.914,62	7.896,61
k	8.103,70	8.057,70	8.039,69	8.021,68	8.003,67	7.985,66	7.967,65
l	8.174,74	8.128,74	8.110,72	8.092,71	8.074,70	8.056,69	8.038,68
m	8.027,67	7.981,67	7.963,66	7.945,65	7.927,64	7.909,62	7.891,61
n	8.174,74	8.128,74	8.110,72	8.092,71	8.074,70	8.056,69	8.038,68
o	8.103,70	8.057,70	8.039,69	8.021,68	8.003,67	7.985,66	7.967,65
**Truncated mersacidin constructs free cysteine control samples (average)**							
**Average** masses (Da)							
**Control samples**	Unmodified		+IAA	+2IAA			
a	7484,305		7.541,38	7.598,45			
c	7465,237		7.522,31	N/A			

S7. Tricine SDS-page of all mutants

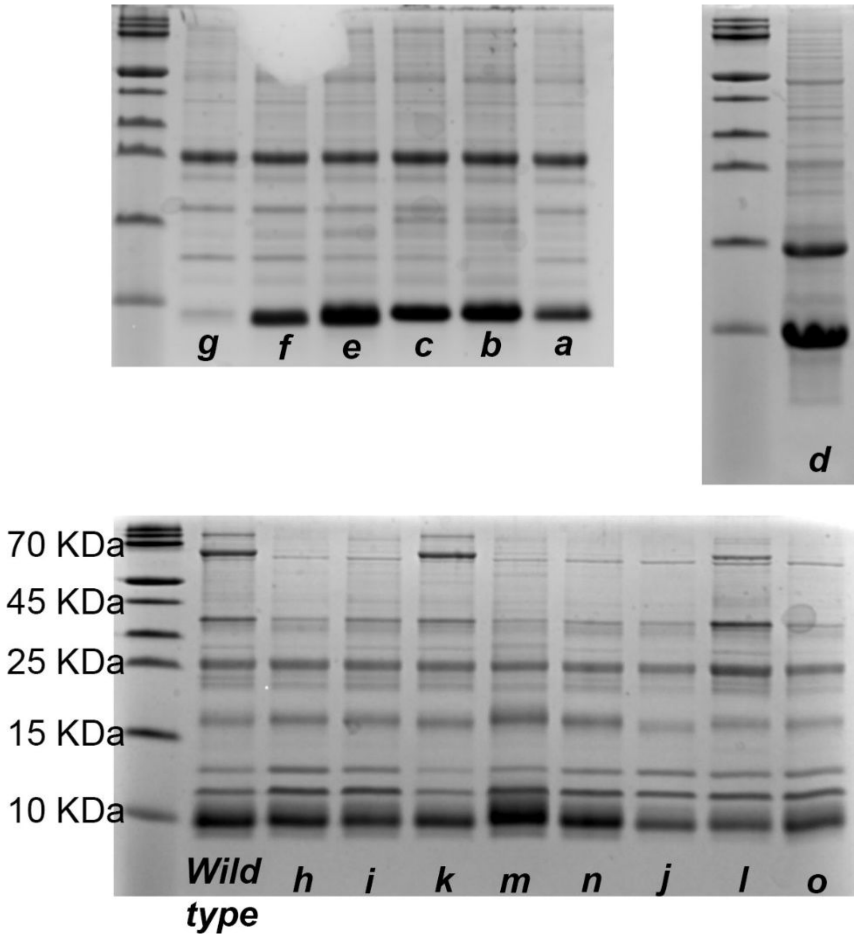

For all samples except *d,* 12 µl of Ni-NTA chromatography elution fraction was loaded with 4 µl of 5 x loading dye. For sample d, 6 µl of Ni-NTA chromatography elution fraction was loaded with 4 µl of 5 x loading dye, because the expression volume was double that of the other mutants. Each slot contains the expression yield of 2 mL of expression culture. As the Wildtype mersacidin is expressed. As the expression yield of wild type mersacidin in the *E. coli* heterologous expression system is around 7.5 mg/L, we can estimate the yield of each individual construct to be between 1 and 15 mg/L of expression volume.

S8. Activity tests all mutants

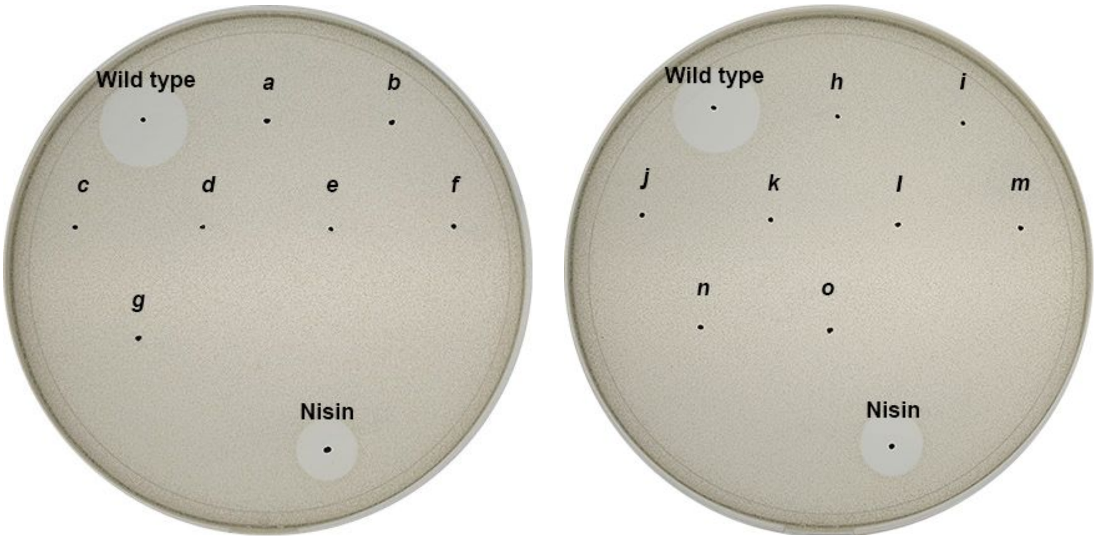

For all mutants, 8 µL of freeze-dried C-18 purified peptide, dissolved in 150 µL Milli-Q water, was digested by adding 1 µL of AprE-His, and setting the final volume to 10 µL by adding 1 µL of Milli-Q water, according to the previously described protocol[2]. For the wild type control, containing MrsMD modified His6-MrsA, 2 µL of freeze-dried C-18 purified peptide, dissolved in 150 µL Milli-Q water was digested by adding 1 µL of AprE-His. For the control, the final volume was set to 10 µL by adding 7 µL of Milli-Q water. A nisin solution of 25 ng/µL was used as a positive control. Of all samples and controls, 9 µL was spotted on the activity plates.

References

(1) Viel, J. H.; Jaarsma, A. H.; Kuipers, O. P. Heterologous Expression of Mersacidin in Escherichia Coli Elucidates the Mode of Leader Processing. *ACS Synth. Biol.* **2021**, *10* (3), 600–608. https://doi.org/10.1021/acssynbio.0c00601.

(2) Viel, J. H.; Kuipers, O. P. Mutational Studies of the Mersacidin Leader Reveal the Function of Its Unique Two-Step Leader Processing Mechanism. *ACS Synth. Biol.* **2022**, *11* (5), 1949–1957. https://doi.org/10.1021/acssynbio.2c00088.
